# Identification and enrichment of human retinal organoid-derived red/green cone-competent precursors with enhanced axon dynamics

**DOI:** 10.1186/s13287-026-05128-9

**Published:** 2026-07-04

**Authors:** Praveen J. Susaimanickam, Elizabeth Capowski, Mai N. Xiong, Kimberly L. Edwards, Yashdeep Phanse, Madalynn J. Welch, Austin Pier, Steven J. Mayerl, Maria A. Fernandez Zepeda, Kyle D. Peterson, M. Joseph Phillips, Divya Sinha, Timothy M. Gomez, David M. Gamm

**Affiliations:** 1 Waisman Center, University of Wisconsin-Madison, Madison, USA; 2 McPherson Eye Research Institute, University of Wisconsin-Madison, Madison, USA; 3 Department of Neuroscience, University of Wisconsin-Madison, Madison, USA; 4 Department of Ophthalmology and Visual Sciences, University of Wisconsin-Madison, Madison, USA

**Keywords:** Pluripotent stem cells, Retinal organoids, Cone-rod reporter line, L/M cones, CD166/ALCAM, Photoreceptor axon extension, Cell replacement therapy, Macular diseases

## Abstract

**Background:**

Cell replacement therapies aimed at restoring foveal vision require a robust source of red/green (long/medium wavelength, or L/M) cone photoreceptors with intrinsic properties conducive to functional integration into host retina. Recent evidence has shown that cones present within mature human retinal organoids (ROs) can generate light responses comparable to macaque foveal cones. However, only cone precursors from early developing ROs possess a capacity for cell-autonomous axonogenesis. Therefore, we sought to identify and enrich for a population of early L/M cone-competent precursors with intrinsically superior axon dynamics that would provide an ideal donor cell source for future foveal reconstruction efforts.

**Methods:**

We developed a dual L/M cone/rod reporter (L/M-CRR) line to unequivocally identify early L/M cone-competent precursors from human ROs. To do so, we used CRISPR/Cas9 to link a tdTomato transgene to the endogenous *THRB2* promoter in the WA09 *NRL*^+/eGFP^ rod reporter line. Differentiated ROs were characterized at early (day 50) and intermediate (day 100) developmental stages using a combination of live fluorescence imaging, immunocytochemistry, and flow cytometry, followed by fluorescence-activated cell sorting and bulk RNAseq analysis to delineate the unique molecular signature of early L/M cone-competent precursors.

**Results:**

THRB2-driven tdTomato fluorescence faithfully demarcated L/M cone-competent precursors throughout RO development, although fluorescence declined in later ROs as L/M cones matured. Transcriptomic profiling revealed that day 50 sorted tdTomato^+^ cells were specifically enriched for genes associated with neural development, axon extension and guidance, and cell migration, which included a gene encoding the cell surface protein CD166/ALCAM. Magnetic-activated cell sorting using an anti-CD166 antibody resulted in specific enrichment of early, highly axonogenic L/M cone-competent precursors assessed by time-lapse imaging.

**Conclusions:**

The L/M-CRR line enables definitive identification and transcriptomic characterization of L/M cone-competent precursors throughout early to mid-stage RO development. Our investigation also revealed that CD166/ALCAM can be used to independently identify and enrich for a subset of early L/M cone-competent precursors that selectively display axon dynamicity conducive for retinal circuit integration. Our studies provide the first insights into early human L/M cone development and establish a method to isolate L/M cone-competent precursors with enhanced axon dynamics, which constitutes a compelling cell population for treating central vision loss caused by photoreceptor degeneration.

**Supplementary Information:**

The online version contains supplementary material available at https://doi.org/10.1186/s13287-026-05128-9.

## Background

The fovea is a small (1.5 mm in diameter), specialized region of the retina with a photoreceptor (PR) population that is nearly exclusively composed of long (red) and medium (green) wavelength cones (L/M cones) [1]. It provides high acuity color vision in humans and nonhuman primates, the only two mammals who possess this structure, and loss of foveal L/M cones through disease or injury results in profound and irreversible central visual impairment. Currently, there are no options to treat late stage foveal degeneration, although some therapeutic strategies are under investigation, including cell transplantation [2–4]. While advancements in human pluripotent stem cell (hPSC) and organoid technologies have made retinal cell replacement feasible [5–7], restoration of foveal vision specifically requires an ample supply of L/M cones, which is challenging to achieve given their scarcity within the retina [8, 9]. Furthermore, a donor L/M cone population should possess the developmental plasticity necessary to survive, integrate, mature, and function in a potentially inhospitable host environment.

Recently, it was shown that L/M cones in aged (> 240 days) hPSC-derived retinal organoids (ROs) can respond to light in a repetitive, graded, and wavelength-specific (i.e., red or green) manner akin to adult macaque foveal cones [10]. A subsequent study revealed that only immature RO-cones (< 80 days of differentiation) were capable of migration and cell autonomous axon extension [11]. Together, these data support an optimal strategy for foveal reconstruction in which early L/M cones are transplanted beneath the central retina and allowed to differentiate to functional maturity in situ. Such an approach carries cost benefits inherent to a shortened cell manufacturing time, but also raises concerns about co-delivery of other early retinal cell types, which might reduce the effective L/M cone dose and create potential barriers to synaptic integration, among other untoward effects [12]. To date, however, no tools or techniques have been developed to identify and selectively harvest L/M cones at their earliest stages of post-mitotic differentiation.

Human pluripotent stem cell (hPSC) reporter lines enable the identification of specific cell lineages in live cultures. They are also valuable for studying the temporal expression profile of the gene to which the reporter is linked, particularly when antibodies that recognize the gene product are unavailable. Multiple photoreceptor (PR) reporter hPSC lines have been generated to study rod and/or cone development and to facilitate their enrichment for in vitro and in vivo studies [12–19]. However, none of the published hPSC-derived cone reporter lines [20–22] are specific to L/M cones, nor do they delineate very early L/M cone-competent precursors. The availability of an hPSC reporter line that marks L/M cones immediately after commitment would not only aid foveal reconstruction efforts, but would also provide a unique tool to investigate mechanisms of human L/M cone fate determination.

There is a wealth of in vivo evidence that the transcription factor thyroid hormone receptor beta 2 (Thrb2) plays a critical role in the establishment of L and/or M cone identity [23–27]. Consistent with this function, Eldred et al. [28] showed that hPSC-ROs can be induced to generate supernumerary L/M cones in vitro at the expense of S cones by adding thyroid hormone early during differentiation. In humans, the *THRB* gene generates two isoforms, THRB1 and THRB2 [29–31], the former of which is essential for proper development and functioning of the brain and auditory system but does not have a known role in cone development in vivo. THRB1 and THRB2 have identical DNA and C-terminal ligand binding domains with unique N-termini due to the presence of distinct promoters [31–34]. The THRB2 N-terminus is conserved across vertebrate species and is encoded by a single exon, but little is understood about the regulatory elements that govern its isoform-specific expression. As such, creation of a THRB2-specific reporter line requires placement of the reporter gene immediately downstream of the endogenous THRB2 promoter. Furthermore, since haploinsufficiency of the T_3_ ligand binding domain (a region common to THRB1 and THRB2) can cause Resistance to Thyroid Hormone (RTH) syndrome in humans [35, 36], it is prudent to retain biallelic expression of both isoforms.

Taking these factors into account, we employed CRISPR-Cas9 technology to introduce a *tdTomato-P2A* transgene upstream of the *THRB2* translation initiation site (TIS), thereby using the endogenous *THRB2* promoter to control tdTomato expression without affecting native THRB2 expression levels. To increase the utility of the line, we inserted the transgene into our previously described WA09 *NRL*^+/eGFP^ rod reporter line [19], thereby creating a dual L/M cone/rod reporter (L/M-CRR). Initial molecular characterization of L/M-CRR ROs was done by live fluorescence imaging, immunocytochemistry (ICC), and flow cytometry (FC). Bulk RNA sequencing and analysis of fluorescence-activated cell (FAC)sorted L/M-CRR ROs at days 50 and 100 revealed that early L/M cone-competent precursors differentially expressed genes associated with nervous system development, axon guidance, and cell migration. Of particular interest was a unique cluster of genes expressed exclusively in early L/M cone-competent precursors that included the cell surface marker CD166 (also known as Activated Leukocyte Cell Adhesion Molecule, or ALCAM) [37]. Magnetic-activated cell sorting (MACS) using a CD166 primary antibody enriched for a subset of early L/M cone-competent precursors that possess an increased capacity for axon extension and migration. Such properties are highly sought after in ongoing efforts to optimize functional integration of hPSC-derived donor L/M cone-competent precursors for the treatment of central vision loss.

## Methods

### Generation of a dual L/M cone/rod reporter (L/M-CRR) hPSC line

CRISPR/Cas9-based gene editing was used to generate a dual L/M-CRR hPSC line by introducing a tdTomato reporter transgene at the 5′ end of the *THRB2* gene in the previously reported WA09 *NRL*^+/eGFP^ hPSC line [19]. A donor plasmid (pBS_5′tdTomato-P2A-THRB2-3′) (Fig. S1A) was constructed using Gibson Assembly^®^ Master Mix (NEB E2611S) and the following 4 DNA fragments: (i) a 711 bp 5′ homology arm corresponding to the genomic region upstream of the *THRB2* translation initiation site (TIS) (Ensembl ID: ENST00000280696.9 THRB-201) (Data S1), (ii) a 990 bp 3′ homology arm corresponding to the genomic region downstream of the *THRB2* TIS (both PCR-amplified from WA09 *NRL*^+/eGFP^ hPSC genomic DNA), (iii) a 1490 bp *tdTomato* transgene fragment PCR-amplified from the pCAG-tdTomato plasmid (Addgene #83029), and (iv) a pBlueScript II SK(-) backbone (Agilent 212206) linearized with EcoRI and NotI. The primers used for amplifying the above fragments contained modifications to remove the tdTomato stop codon in the amplicon and to add sequences to ensure DNA fragment overlap for homology-mediated directional assembly and also to add a P2A sequence immediately after the *tdTomato* transgene. For single guide RNA (sgRNA) plasmid construction, forward (5′ CACCGGTAAACTAGAAACTGAACCA 3′) and reverse oligos (5′ AAACTGGTTCAGTTTCTAGTTTACC 3′) were annealed and cloned into BsmBI-digested lentiCRISPR v2 plasmid (Addgene 52961) as described in Sanjana et al. [38].

To generate the hPSC L/M-CRR line, WA09 *NRL*^+/eGFP^ hPSCs were dissociated and electroporated with the donor plasmid (25 µg) and sgRNA plasmid (15 µg) as described in Chen et al. [39] and plated on mouse embryonic fibroblasts (MEFs) in human embryonic stem cell medium (hESCM; DMEM/F12 (Gibco 11330-057), 1× MEM nonessential amino acids (MEM NEAA; Gibco 11140-076), 0.5× GlutaMAX™ (Gibco 35050079), 0.1 mM β-mercaptoethanol (Sigma M3148-100ML), and 4 ng/mL bFGF (WiCell)) containing 1 µM Rho Kinase (ROCK)-inhibitor (Calbiochem, H-1152P). To select for cells that received the sgRNA plasmid (lentiCRISPR v2) and to induce higher editing efficiency, cells were treated with 0.33 µg/mL puromycin (Invitrogen, ant-pr-1), at 24-, 48- and 72-hours post-electroporation. Concurrent with puromycin treatment, the cells were fed with MEF-conditioned hESCM containing 1 µM ROCK inhibitor. After removal of the puromycin at 96 hours, cells were cultured in MEF-conditioned hESCM until colonies were visible. Screening of clones was performed by manually selecting single-cell colonies and isolating genomic DNA from a portion of these colonies using QuickExtract DNA Extraction Solution (BIOSEARCH TECHNOLOGIES QE09050). Genotyping was performed with a left junction primer upstream of the 711 base pair 5′ homology arm (Data S1) and a reverse primer interior to the *tdTomato* transgene, allowing specific amplification of the partial *tdTomato* sequence only when it was present in the *THRB2* locus. PCR products were identified via agarose gel electrophoresis and purified using a Zymoclean Gel DNA Recovery Kit (ZYMO RESEARCH D4001). Clones were submitted to Quintara Biosciences for Sanger sequencing to verify proper *tdTomato-P2A* transgene insertion. The resulting two hPSC clones (L/M-CRR C24 and L/M-CRR C38) were expanded and off-target mutation screening was conducted for the ten highest likelihood sites for the sgRNA used in this study. No off-target indels were found at these sites for both clones (Data S2). Targeted incorporation and heterozygosity of the *THRB2* transgene in L/M-CRR C24 and L/M-CRR C38 were validated by PCR using genotyping primers. Both clones were karyotyped and examined for pluripotency marker expression by immunocytochemistry (ICC).

### hPSC lines used in the study and their maintenance in culture

The following hPSC lines were used in the study: WA09 L/M-CRR (generated in this study), unmodified WA09 (WiCell), WA09 *NRL*^+/eGFP^ [19], WA09 *CRX*^+/tdTomato^ [18], and E4-H7 [40]. hPSCs were maintained in 6-well plates on Matrigel^®^ (Corning^®^ 354230) under humidified conditions at 5% CO_2_ and 37 °C using StemFlex (Gibco A3349401) or mTeSR™ Plus medium (STEMCELL TECHNOLOGIES 100–0276) and passaged using Versene (Gibco 15040066) or ReLeSR™ (STEMCELL TECHNOLOGIES 100–0483).

### hPSC-retinal organoid (RO) differentiation

hPSCs were differentiated to ROs using a previously published protocol [8]. Briefly, pluripotent stem cell colonies were detached from cell culture plates to form embryoid bodies (EBs) on day (D) 0. EBs were transitioned from StemFlex (Gibco A3349401) or mTeSR™ Plus medium to neural induction medium (NIM; DMEM/F12, 1% [v/v] N2 supplement (Gibco 17502048), 1× MEM NEAA, 1× GlutaMAX™ and 2 µg/ml heparin (Sigma H-3149)) over the course of 4 days. On D6, media was replaced with fresh NIM supplemented with 12.5 ng/mL Bone Morphogenetic Protein 4 (BMP4; R&D Systems 314-BP-01 M) and the EBs were plated on Matrigel^TM^-coated 6-well plates. Half the media was replaced with fresh NIM on D9, D12, and D15, and on D16 the media was changed to Retinal Differentiation Medium (RDM; 3.5:1.5 DMEM: F12 (Gibco 11965-118 and 11765070, respectively), 2% B27 supplement (Gibco 17504044), 1x MEM NEAA, 1x Antibiotic-Antimycotic (Gibco 15240112), and 1× GlutaMAX™). Between D25-D30, optic vesicle-like colonies became visually apparent by brightfield microscopy and were dissected with a 30-degree stab knife (AMBLER SURGICAL 7536). The resulting free-floating ROs were maintained in poly-HEMA-coated T25 flasks (Sigma P3932, Falcon 353109) with twice-weekly feeding of 3D-RDM (3.5:1.5 DMEM: F12, 2% B27 supplement, 1× MEM NEAA, 1× Antibiotic-Antimycotic, 1× GlutaMAX™, 5% FBS (WiCell), 100 µM taurine (Sigma T0625-10G), and 0.4% [v/v] Chemically Defined Lipid Concentrate (Gibco 11905031)). Live RO cultures were imaged on a Nikon Ts2-FL microscope equipped with a DS-Fi3 camera or a Nikon ECLIPSE Ti2-E equipped with a multi-wavelength LED Light Engine.

### RO dissociation

ROs at designated time points or ranges were dissociated using 10X TrypLE™ Select Enzyme (ThermoFisher Scientific A1217703; 1 mL per 7–10 ROs) for 30–35 min at 37 °C followed by addition of 0.05 mg/mL DNase I Solution (STEMCELL Technologies 100–0762; 2 mL for each mL of 10X TrypLE Select used) and incubation at RT for 15 min. Dissociated ROs were then filtered through a pre-wet 37 μm cell strainer (STEMCELL Technologies 27215) to obtain a single cell suspension. Cell counts were obtained on a Countess 3FL Automatic Cell Counter prior to proceeding with either reverse transcription quantitative real-time PCR (RT-qPCR), flow cytometry (FC), or magnetic-activated cell sorting (MACS).

### RT-qPCR analysis

WA09 ROs on D21, D35, D50, and D100 of differentiation were dissociated as described above into single cells to prepare aliquots of 1 × 10^5^ cells in 1.5 mL microfuge tubes. cDNA was synthesized using the SYBR Green Fast Advanced Cells-to-CT Kit (ThermoFisher Scientific A35380) and qPCR was performed according to manufacturer’s instructions using the primer sets listed. To calculate C(q)s, the Ct values for the genes *THRB2*,* ARR3*,* GNGT2*, and *GNAT2* were normalized by subtracting the geometric mean Ct values of two housekeeping genes (*GAPDH* and *YWHAZ*). Thereafter, cone gene expression levels were quantified relative to the housekeeping genes (set at 1) using the 2(-Delta Delta C(T)) method [41].

To evaluate differences in gene expression across cone photoreceptor-specific genes at different time points, we conducted a two-way analysis of variance (ANOVA) with gene group (*THRB2*, *ARR3*, *GNGT2*, *GNAT2*) and time points (D21, D35, D50, D100) as fixed factors, including their interaction. Assumptions of normality and variance were assessed, where a log-transformation was applied to stabilize normality and variance, justified with Levene and Shapiro-Wilk tests. Planned contrasts were specified to test whether each Group x Time geometric mean differed significantly from *THRB2*, log(1) = 0, using two-sided tests. Multiple comparisons were adjusted using the Bonferroni correction to control Type I error across contrasts. Statistical tests were performed using R software (version 4.4.0) and statistical significance was defined as *p* < 0.05.

### Flow cytometry (FC)

L/M-CRR ROs were processed for FC as described [42]. In brief, L/M-CRR ROs (*n* = 5 independent differentiations; ≥10 ROs per differentiation) at designated time periods (D30-D200) were dissociated as described above (see ‘RO Dissociation’) to obtain single cell suspensions. The cell suspensions were then either subjected directly to FC or to magnetic-activated cell sorting (MACS) followed by FC (see MACS method section). FC was performed using a ThermoFisher Attune NxT and data was analyzed using FlowJo software (Version 10.8.1). Appropriate compensation and fluorescence-minus-one (FMO) controls were used to establish unbiased gates. Integrated Median Fluorescence Intensity (iMFI) was calculated as the product of the percentage of tdTomato-positive cells and their corresponding median fluorescence intensity. Statistical significance was assessed using the One-Way ANOVA post hoc Tukey’s multiple comparisons test in Graph pad Prism (version 10.2.3).

### Magnetic-activated cell sorting (MACS) using anti-CD166

Singularized cells from L/M-CRR ROs at D50 were incubated with Biotin-conjugated ALCAM/CD166 (Miltenyi Biotec 130-126-100; 1:50 dilution) for 20 min at 4 °C and washed three times with MACS Separation buffer (autoMACS Rinsing Solution (Miltenyi Biotec 130-091-222) containing 0.5% MACS BSA Stock Solution (Miltenyi Biotec 130-091-376)). Thereafter, cells were incubated with Anti-Biotin microbeads (Miltenyi Biotec 130-090-485; 1:10 dilution) and passed through an LS column (Miltenyi Biotec 130-042-401) placed on a magnetic stand. Cells that flowed through the column were collected as the ALCAM/CD166-negative fraction. The LS column with bound cells was washed thrice with MACS Separation Buffer and the flow-through was added to the ALCAM/CD166 negative fraction. Thereafter, the column was removed from the magnetic stand and the remaining cells were washed from the column to obtain the ALCAM/CD166-positive fraction. Cells from the unsorted, ALCAM/CD166-positive and ALCAM/CD166-negative cell populations were either immediately subjected to FC-based quantification or resuspended in 3D-RDM containing 10 µM ROCK inhibitor and plated at a density of 100,000 cells/well on optical-grade 96-well plates (Nunc 152040) coated with 50 µg/mL mouse laminin (Gibco 23017015). A complete media change was performed the next day and every other day thereafter with fresh 3D-RDM until D7, at which time they were fixed for ICC. Statistical significance of FC-based quantifications was assessed using one-way ANOVA with Tukey’s post hoc test in Graph pad Prism (version 10.2.3).

### Immunocytochemistry (ICC)

hPSC monolayers were cultured on Matrigel^®^-coated optical grade 96-well plates. hPSC monolayers and plated cells from dissociated ROs were fixed using 4% paraformaldehyde (PFA) (Electron Microscopy Sciences 15710) for 20 min at RT, and washed with 1X PBS (Fisher bioreagents BP2438-20). Intact ROs were fixed in 4% PFA at RT with gentle agitation for 1 h and rinsed with 1X PBS. ROs were cryoprotected first in 15% sucrose and then in 30% sucrose over a time span of 1–3 h or until ROs sunk. Cryoprotected ROs were then embedded in OCT freezing medium (Fisher Scientific 72592) and cryosectioned at a thickness of 15 μm at -25 °C and mounted on Superfrost Plus slides (ThermoFisher Scientific 12-550-15). Fixed hPSC monolayers and/or cryosectioned ROs were blocked in blocking solution (10% normal donkey serum (Sigma-Aldrich S30-100ML), 5% BSA (Sigma-Aldrich A7906-100G), and 0.5% Triton X-100 (Sigma-Aldrich X100) in 1X PBS) for 1 h at RT and incubated overnight at 4 °C with primary antibodies diluted in blocking solution. For a list of primary and secondary antibodies with concentrations used. Samples were washed with 1X PBS and incubated at RT for 35 min (protected from light) with 1:500 diluted fluorophore-conjugated secondary antibodies directed against respective primary antibody hosts. Samples were washed with 1X PBS and immunostained RO sections were mounted with ProLong Gold Antifade with DAPI (ThermoFisher Scientific P36941). Counterstaining of nuclei in hPSC monolayers and plated cells from dissociated RO cells was performed by including DAPI (Invitrogen D1306; 1:5000 dilution) in the secondary antibody mix. Immunostained hPSC monolayers and plated RO cells were stored in 1X PBS at 4 °C and imaged within 2 days. All samples were imaged using either a Nikon A1R-Si laser scanning confocal microscope or a Leica Stellaris 8 confocal system.

### Bulk RNA sequencing and data processing

D50 and D100 L/M-CRR ROs were dissociated into single cells using the Papain Dissociation System (Worthington Biochemical Corporation LK003150) according to the manufacturer’s protocol. Cells were then sorted into the following groups using a BD FACSAria housed in a biosafety cabinet: (1) D50 THRB2:tdTomato^–^ ‘other’ retinal cells, (2) D50 THRB2:tdTomato^+^ early L/M cone-competent precursors, (3) D100 THRB2:tdTomato^–^/NRL:eGFP^–^ ‘other’ retinal cells, (4) D100 THRB2:tdTomato^+^ late L/M cone-competent precursors, and (5) D100 NRL:eGFP^+^ early rod precursors (Data S3). RNA was isolated from each sample using the RNAeasy mini spin kit (Qiagen 74104) according to the manufacturer’s instructions and libraries for sequencing were prepared using the TruSeq Stranded Total RNA Library Prep kit (illumina 20020599) for humans. Libraries were sequenced on a NovaSeq6000 and 30 million 2 × 150 nucleotide reads were collected for each sample.

Bioinformatic analysis of transcriptomic data adhered to recommended ENCODE guidelines and best practices for RNA-Seq (https://www.encodeproject.org/about/experiment-guidelines/). Alignment of adapter-trimmed (Skewer v0.1.123 [43]) 2 × 150 (paired-end; PE) bp strand-specific Illumina reads to the *Homo sapiens* GRCh38.p10 genome (assembly accession NCBI: GCA 000001405.25) was achieved with the Spliced Transcripts Alignment to a Reference (STAR v2.5.3a) software [44]. Expression estimation was performed with RSEM v1.3.0 [45] to obtain expected read count and Transcripts Per Million (TPM) values. To test for differential gene expression among individual group contrasts, expected read counts obtained from RSEM were used as input into edgeR [46] (v3.16.5). Inter-sample normalization was achieved with the trimmed mean of M-values (TMM) [47] method. Statistical significance of the negative-binomial regression test was adjusted with a Benjamini-Hochberg FDR correction at the 5% level [48]. Prior to statistical analysis with edgeR, genes with low expression were removed using independent filtering with the filterByExpr function. This function retains genes that have sufficiently large counts, defined as a count-per-million (CPM) value corresponding to at least 10 raw counts in the smallest library size, in a minimum number of samples. The minimum number of samples is set to the number of biological replicates in the smallest experimental group, ensuring that retained genes have enough power for detection. The validity of the Benjamini-Hochberg FDR multiple testing procedure was evaluated by inspection of the uncorrected p-value distribution. Multidimensional scaling plots, volcano plots, and heatmaps were generated using R (version 4.4.0). TPM values were log-2 transformed (log2(TPM) + 1) and z-score normalized across samples. K-means clustering was performed with K = 6 clusters and visualized using the R package ComplexHeatmap [49]. Gene ontology analysis was performed using Enrichr [50] and Gene Family identification was done using GSEA-Molecular Signatures Database, MsigDB [51].

### Image-based cell counting

Large images (at least 1.5 × 1.5 mm) of immunostained sections of D50 L/M-CRR ROs were obtained using a Nikon ECLIPSE Ti2-E equipped with a multi-wavelength LED Light Engine. Using ImageJ software (version 1.54p), RO regions were demarcated by drawing a segmented line between the densely packed outer (apical) nuclei and comparatively sparse inner (basal) nuclei (Fig. S7). The cell counter plugin was used to record the manually counted red-only cells, green-only cells and red+green cells. Statistical significance was determined by an unpaired t-test using Graph Pad Prism (version 10.2.3).

### Axon terminal dynamicity measurement

Axon terminal dynamicity measurements on tdTomato-expressing PRs were accomplished as previously reported [11]. Briefly, unsorted and CD166-positive cells (i.e., positive fraction from CD166 MACS) from WA09 CRX^+/tdTomato^ D50 ROs were plated at 50,000 cells/cm^2^ on poly-D-Lysine coated glass-bottom dishes. After cell adherence, the culture dishes were flooded with 2 mL RDM and maintained for 2–4 days prior to imaging. For live imaging, a gas-impermeant chamber was created by affixing a glass ring to the glass-bottom dish (10–20 mm diameter) with vacuum grease (Dow Corning #Z273554), filling it with culture media (RDM, low phenol red), and sealing the chamber with a coverslip. During live imaging, the dishes and stage were kept at 37 °C with a Zeiss Incubator XLmulti S1. Images were captured using a Zeiss LSM 800 inverted microscope with either a Plan Apo 63x oil immersion objective (NA 1.4) or a Plan Apo 20x air objective (NA 0.8).

For quantification of axon terminal dynamicity, time-lapse images were acquired for 15 min (at 3-min intervals). Axon terminal dynamics were analyzed using ImageJ (v1.52p) as described in detail by Rempel et al. [11]. Briefly, for each axon, the distal growth cone (axon terminal) region was manually selected and cropped from the time-lapse series. A macro (ImageJ script that automates repetitive image-processing steps) was used to convert each tdTomato image into a binary black-and-white image (or mask) in which pixels representing the growth cone are assigned a value of 1 (white) and background pixels are assigned a value of 0 (black). The binary masks of consecutive time-lapse images were then compared frame-by-frame by subtraction (Frame X + 1 minus Frame X). This produced a difference image in which pixels appearing between frames represent growth cone extension and pixels disappearing between frames represent growth cone retraction. Extended and retracted areas were quantified based on pixel counts of the difference images for each 3-minute interval. Axon terminal dynamicity was then calculated as the sum of the extended and retracted areas, normalized to the mean growth cone area across the corresponding frames.

For extended PR axon length measurements, calibrated scale bars were used to set the pixel-to-micron ratio and the extended neurite lengths were measured by tracing the neurites using the freehand tool of ImageJ. Neurite lengths were calculated as the distance between the cell body centroid and the terminal centroid. For both axon dynamicity and axon length measurements, statistical significance was determined using unpaired t-tests. For fluorescence intensity (FI) measurements, cells were fixed and immunostained for CD166/ALCAM and tdTomato as described above (see ‘Immunocytochemistry’), and dye-conjugated phalloidin (Invitrogen A22287) was used to label F-actin. FI for ALCAM/CD166 and F-actin was measured by thresholding on the tdTomato signal, cropping the ROI so that only the terminal was included, and measuring the mean intensity. Statistical significance for fluorescence intensity measurements was assessed using one-way ANOVA with Tukey analysis Graph Pad Prism (version 10.2.3).

### Statistical analyses

Statistical analyses were conducted using appropriate tests as detailed above in their corresponding methods sections. All error bars indicate standard deviation (SD).

## Results

### Generation of a dual L/M cone/rod reporter (L/M-CRR) line

**Fig. 1:**
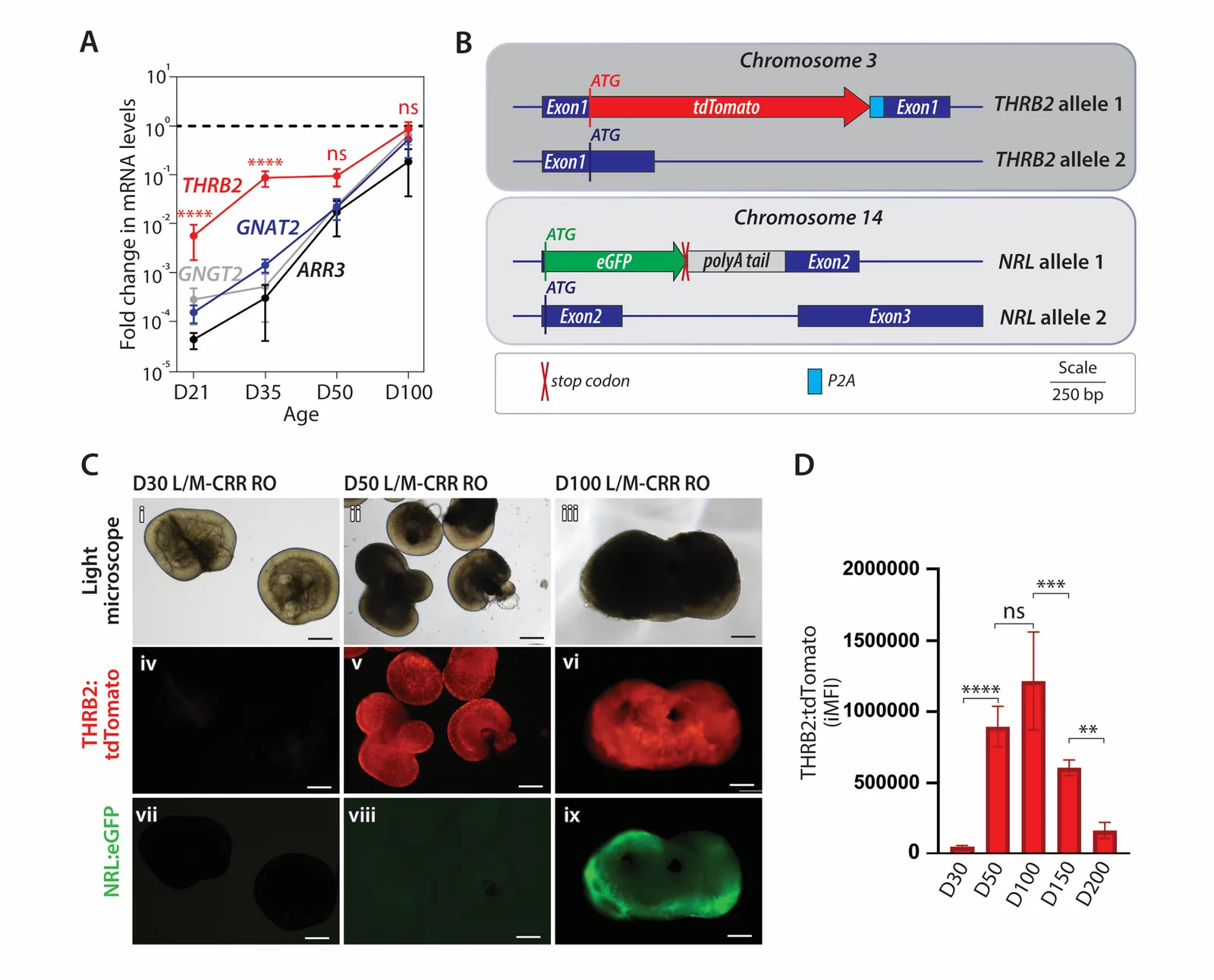
Generation of a dual L/M cone/rod reporter (L/M-CRR) line. **A** Graph showing the fold change in mRNA expression levels of *THRB2* and selected cone-specific genes compared to the geometric mean of two housekeeping genes (*GAPDH* and *YWHAZ*, dashed line at 1) in WA09 hPSC ROs (*n* = 3) from D21 to D100. **B** Schematic depicting the insertion of a *tdTomato-P2A* reporter gene construct at the ATG translation initiation site of one *THRB2* allele in the existing WA09 *NRL*^+/eGFP^ rod reporter line to create a dual L/M cone and rod reporter (L/M-CRR) line. Note that this strategy renders only the *NRL* gene heterozygous, which is inconsequential in humans due to haplosufficiency. **C** Light microscopic images (i-iii), tdTomato fluorescence images (reflecting *THRB2* expression) (iv-vi), and eGFP fluorescence images (reflecting *NRL* expression) (vii-ix) of live L/M-CRR ROs at D30, D50, and D100. THRB2:tdTomato live fluorescence is first detected between D35-D40, whereas NRL:eGFP fluorescence appears between D70-D80 (data not shown). **D** Bar graphs showing integrated Median Fluorescence Intensity (iMFI) of THRB2:tdTomato expression quantified by flow cytometry (FC) in L/M-CRR ROs at D30-D200 (> 10 ROs for each time point, *n* = 5 independent differentiations). Graphs in panels **A** and **D**, are plotted as mean ± SD. *P < 0.05, **P < 0.01, ***P < 0.001, ****P < 0.0001, ns = non-significant (Graph **A** Two-Way ANOVA with gene group and time point, Graph **D** One-Way ANOVA with Tukey analyses). Scale bars for panel **C** 200 μm

To verify that *THRB2* is highly expressed at early time points during RO differentiation, we compared its expression profile to those of other cone-specific genes used in hPSC cone reporter lines (*GNAT2*, *GNGT2*, and *ARR3*) [20–22] at D21, D35, D50 and D100 (Fig. 1A). *THRB2* mRNA expression was significantly higher at D21 and D35 compared to *GNAT2*, *GNGT2* and *ARR3*. At D50 and D100, no significant differences in expression levels were found among any of the cone-specific genes, although *THRB2* trended higher at D50. These findings demonstrate that *THRB2* expression precedes that of other cone genes, supporting its use as a candidate to drive reporter gene expression in cones at their earliest stages of differentiation. Furthermore, unlike *GNAT2*, *GNGT2*, and *ARR3*, which are present in all cone subtypes, *THRB2* is specific to L/M cones and crucial for their development in all species examined to date [23–25, 27, 29, 52–58].

CRISPR-Cas9 gene editing was used to create the L/M cone/rod reporter (L/M-CRR) line by introducing a *tdTomato-P2A* transgene at the translation initiation site (TIS) of one endogenous *THRB2* locus (Data  S1) within the previously reported WA09 *NRL*^+/eGFP^ rod reporter line [19] (Fig. 1B), enabling regulated expression of both tdTomato and THRB2 by the endogenous *THRB2* promoter. Two clones (C24 and C38) demonstrated targeted incorporation of the *tdTomato* reporter transgene at a single *THRB2* locus. These clones were expanded and off-target mutation screening on the ten highest-likelihood sites for the sgRNA showed no off-target indels in either clone (see Data S2). Both gene-edited dual L/M-CRR clones were then karyotyped and analyzed to confirm absence of chromosomal abnormalities and presence of essential pluripotency marker.

**Fig. 2:**
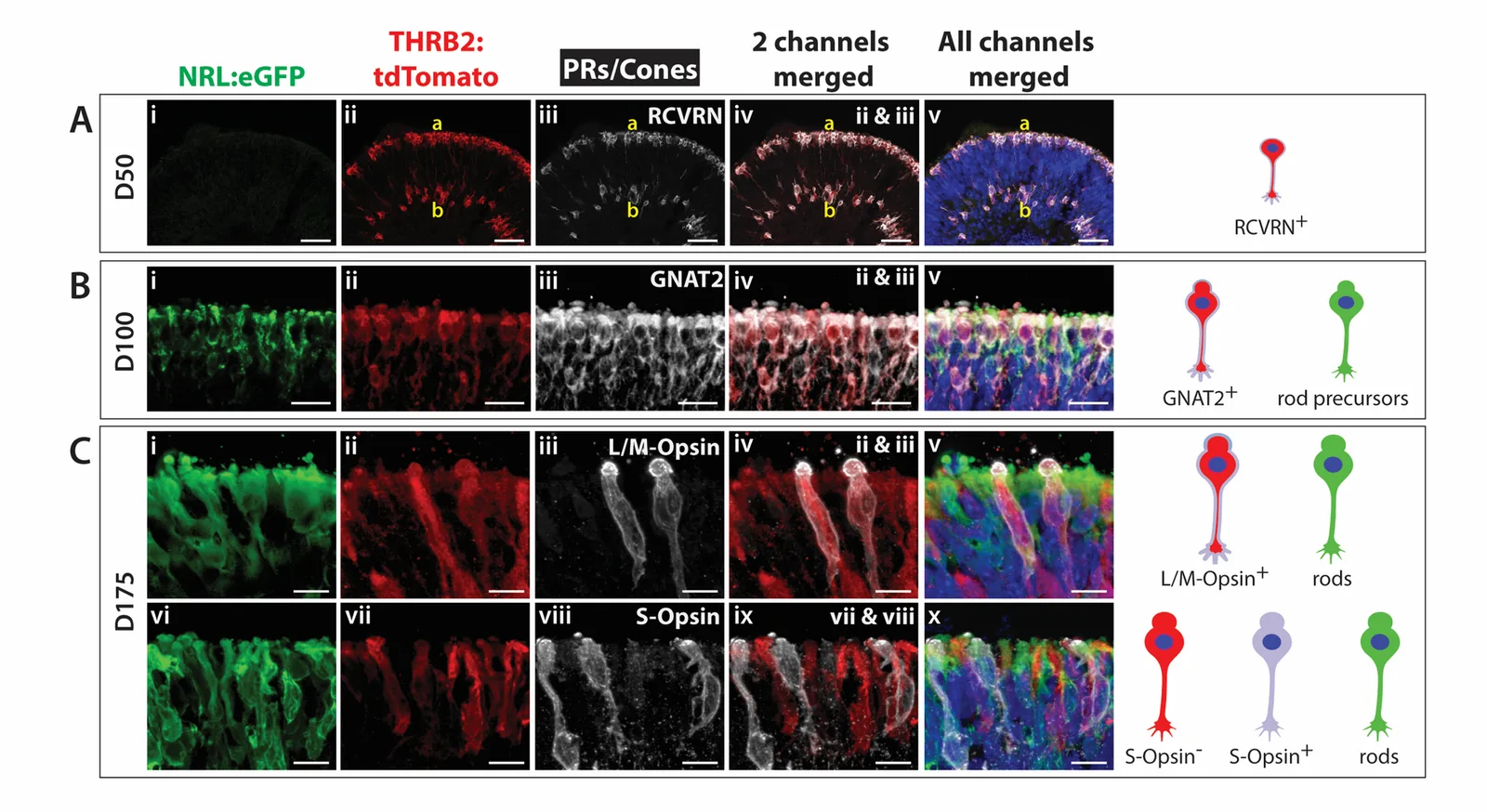
THRB2:tdTomato is expressed exclusively in L/M cone-competent precursors. ICC images of **A** D50 L/M-CRR ROs showing co-expression of THRB2:tdTomato (red) (ii, iv, v) and the pan-PR marker RCVRN (white) (iii-v) in cells along the apical (a) and basal (b) borders of the developing neuroepithelium. The absence of NRL:eGFP expression (green) is also shown (i, v). **B** Cells expressing THRB2:tdTomato (red) (ii, iv, v) within the developing PR layer of D100 L/M-CRR ROs co-express the cone marker GNAT2 (white) (iii-v), but do not co-express the rod marker NRL:eGFP (green) (i, v). **C** Cells expressing THRB2:tdTomato (red) (ii, iv, v, vii, ix, x) in D175 L/M-CRR ROs co-express the mature L/M cone marker L/M-Opsin (white) (iii-v) but not the mature S-cone marker S-Opsin (white) (viii, ix, x) or the rod marker NRL:eGFP (green) (i, v, vi, x). **A** (iv), **B** (iv), and **C** (iv, ix) are merged images of red and white channels at D50, D100, and D175, respectively. **A** (v), **B** (v), and **C** (v, x) are merged images of all channels (including nuclei stained with DAPI in blue) at D50, D100, and D175, respectively. Scale bars for panels **A** 50 μm, **B** 25 μm, and **C** 10 μm

Retinal organoids (ROs) were differentiated from both L/M-CRR clones (C24 and C38) according to our previously described protocol [8] and characterized via live fluorescence imaging (Fig. 1C), flow cytometry (Fig. 1D) and immunocytochemistry (Fig. 2). Cells expressing THRB2:tdTomato were first observed in the basal neuroblastic layer of L/M-CRR ROs at D30 by ICC and slightly later (~ D35) by live fluorescence imaging. Thereafter, THRB2:tdTomato^+^ cells increased in abundance through D100 (D50 and D100 shown in Fig. 1C i-vi). Flow cytometric (FC) quantification of THRB2:tdTomato in L/M-CRR ROs between D30-D200 confirmed an increase in integrated median tdTomato fluorescence intensity through D100, followed by a sharp decrease to near absent levels by D200 (Fig. 1D). As reported previously by our group [19], rod precursors expressing NRL:eGFP were first observed at ~D70 (not shown) and became prevalent by D100 (Fig. 1C vii-ix). Unlike THRB2:tdTomato, NRL:eGFP expression persisted indefinitely in L/M-CRR ROs, also consistent with published findings [19]. Of note, THRB2:tdTomato fluorescence was distributed throughout ROs by live imaging, whereas NRL:eGFP fluorescence was present almost exclusively at or near their surface (Fig. 1C v-vi vs. ix).

### THRB2:tdTomato is expressed in L/M-Opsin^+^ cone-competent precursors but not in rods or S-Opsin^+^ cones

In the absence of isoform-specific THRB2 antibodies, we sought to determine whether THRB2:tdTomato exclusively labels developing L/M cone PRs in L/M-CRR ROs by examining tdTomato expression over time relative to available PR and cone markers, NRL:eGFP, and S– and L/M-Opsin. At D50, prior to the expression of rod or cone subtype-specific markers, THRB2:tdTomato^+^ cells universally co-labeled with the pan-PR marker, Recoverin (RCVRN), broadly indicating an early PR identity (Fig. 2A). Of additional note, THRB2:tdTomato^+^ cells at D50 were predominantly found at or between the apical and basal borders of the RO neuroepithelium, which corresponds to the radial migratory range of developing PRs. By D100, early rod PRs expressing NRL:eGFP were prevalent, almost none of which co-expressed tdTomato. In contrast, all D100 and D175 THRB2:tdTomato^+^ PRs co-labeled with the pan-cone marker GNAT2 (Fig. 2B). Additionally, THRB2:tdTomato did not co-express with markers of non-photoreceptor retinal cells at D100 and D160, time points when ROs contain a maximum diversity of cell types [8]. By D175, S and L/M cones can be delineated in ROs via expression of S- or L/M-Opsin, respectively (Fig. 2C). Although THRB2:tdTomato expression is in decline by this stage of PR maturation (Fig. 1D), the remaining THRB2:tdTomato^+^ cells co-labeled only with L/M-Opsin and not S-Opsin or NRLeGFP (Fig. 2C). Image-based quantification of D175 L/M-CRR RO sections immunostained with L/M-Opsin (*n* = 5) and S-Opsin (*n* = 5) confirmed that all of the L/M-Opsin-expressing cells (*n* = 70) and none of the S-Opsin-expressing cells (*n* = 35) expressed THRB2:tdTomato (Fig. 2C, Fig. S5A-C). Thus, L/M-CRR ROs exclusively and independently label L/M cones and rods.

### Transcriptome profiling of L/M cone-competent and rod precursors

**Fig. 3:**
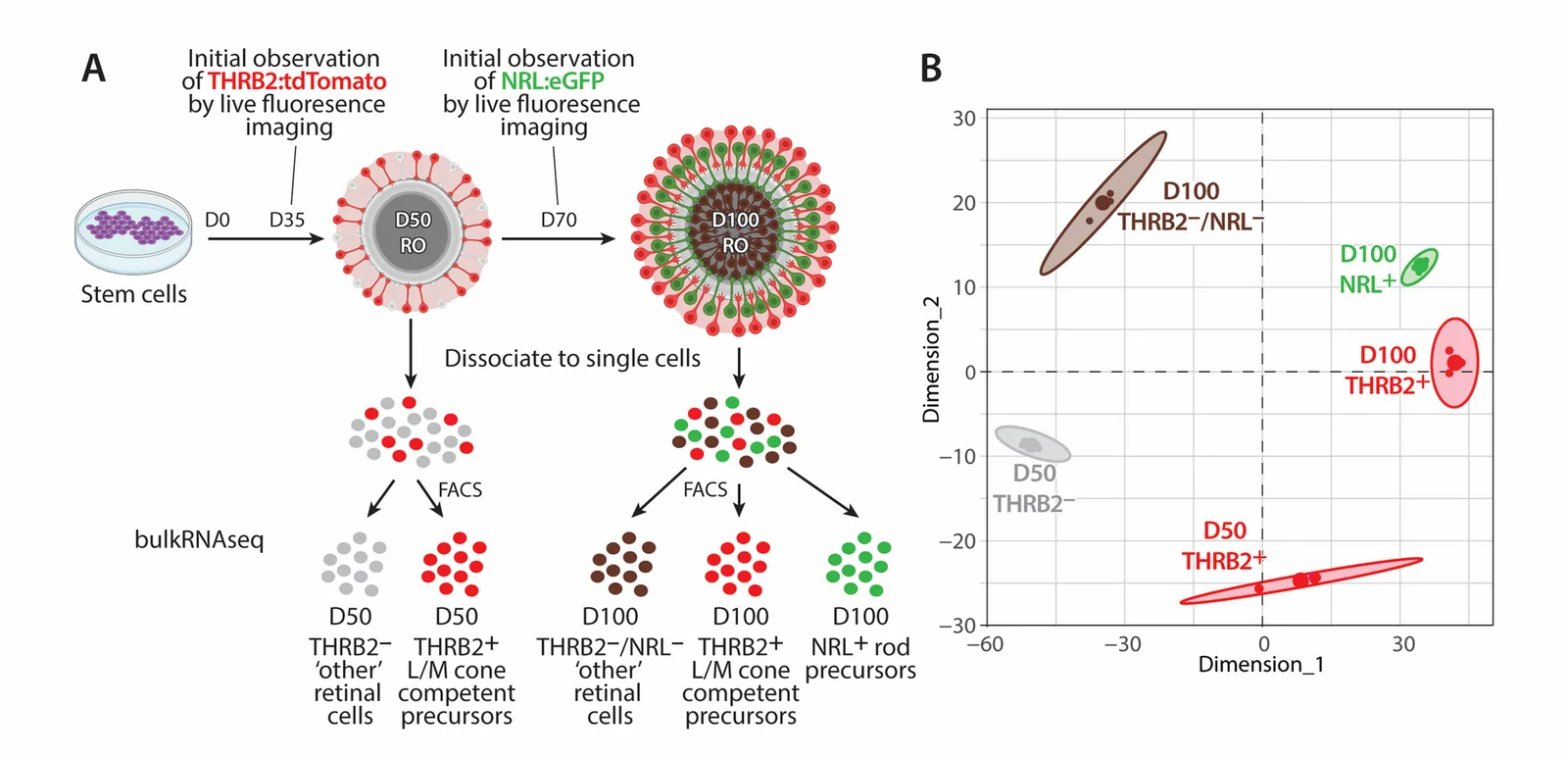
Workflow and quality control analysis for bulk RNA sequencing of L/M-CRR ROs. **A** Schematic representation of the workflow for bulk RNA sequencing. In L/M-CRR ROs, endogenous *THRB2* promoter-controlled tdTomato expression begins ~D35 and endogenous NRL promoter-controlled eGFP expression begins ~D70. L/M-CRR ROs were dissociated into single cells at D50, when THRB2:tdTomato^+^ L/M cone-competent precursors are present but not NRL:eGFP^+^ rod precursors, and D100, when both THRB2:tdTomato^+^ and NRL:eGFP^+^ populations are present. Dissociated cells were FAC-sorted prior to bulk RNA sequencing to generate the following cell populations: (1) D50 THRB2:tdTomato^–^ ‘other’ retinal cells, (2) D50 THRB2:tdTomato^+^ early L/M cone-competent precursors, (3) D100 THRB2:tdTomato^–^/NRL:eGFP^–^ ‘other’ retinal cells, (4) D100 THRB2:tdTomato^+^ late L/M cone-competent precursors, and (5) D100 NRL:eGFP^+^ early rod precursors. **B** Multidimensional scaling plot showing that the gene expression profiles of the biological replicates (*n* = 4) from each cell population cluster tightly together. The larger colored dots and ellipses indicate the respective mean value and 95% confidence interval of each group

To characterize the transcriptomes of L/M cone-competent precursors at D50 and D100, rod precursors at D100, and remaining ‘other’ retinal cells at D50 and D100, four independent differentiations of L/M-CRR ROs at D50 and D100 (minimum of 100 ROs per differentiation) were dissociated into single cells and subjected to fluorescence activated cell (FAC)-sorting to isolate the following cell populations: (1) D50 THRB2:tdTomato^–^ ‘other’ retinal cells, (2) D50 THRB2:tdTomato^+^ early L/M cone-competent precursors, (3) D100 THRB2:tdTomato^–^/NRL:eGFP^–^ ‘other’ retinal cells, (4) D100 THRB2:tdTomato^+^ late L/M cone-competent precursors, and (5) D100 NRL: eGFP^+^ early rod precursors (Fig. 3A , Data S3). RNA from each cell population was isolated and sequenced, which revealed highly similar transcriptomic profiles within biological replicate (*n* = 4) sample groups (Fig. 3B).

### Identification of genes selectively upregulated in D50 L/M cone-competent precursors

**Fig. 4:**
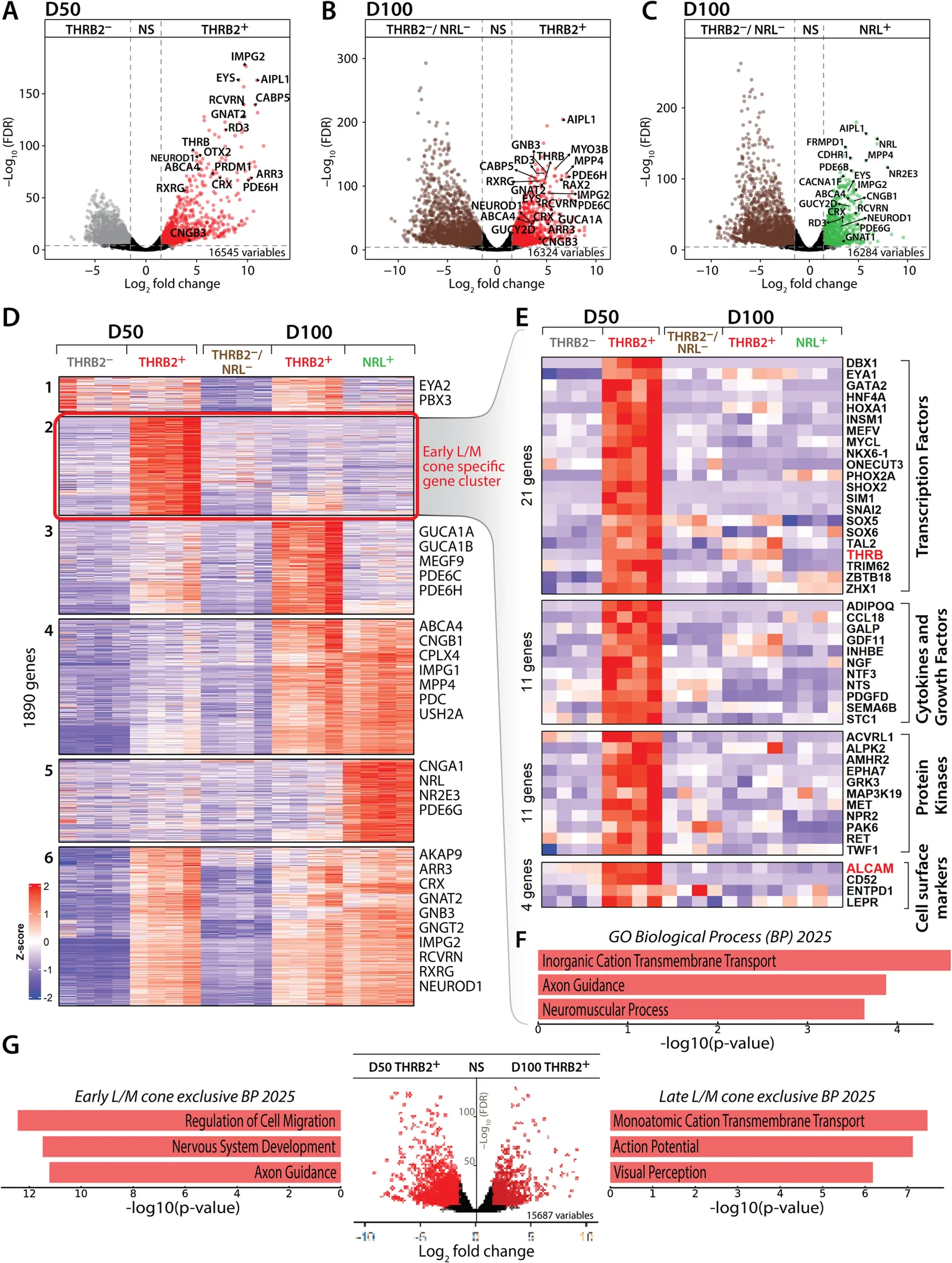
Bulk RNAseq analysis of dissociated and FAC-sorted L/M-CRR ROs at D50 and D100. **A**–**C** Volcano plots showing differentially expressed genes from the following FAC-sorted bulk RNAseq analyses: **A** D50 THRB2^+^ cells vs. D50 THRB2^–^ cells, **B** D100 THRB2^+^ cells vs. D100 NRL^–^/THRB2^–^ cells, and **C** D100 NRL^+^ cells vs. D100 NRL^–^/THRB2^–^ cells. Selected upregulated genes are shown. **D** Heatmap showing 1890 upregulated genes in D50 THRB2^+^ cells, D100 THRB2^+^ cells, and/or D100 NRL^+^ cells (4 biological replicates for each sample group. *n* = 4). K means clustering (K = 6) reveals a unique cluster (Cluster 2; demarcated by the red box) of 308 genes selectively upregulated in the D50 THRB2^+^ L/M cone-competent precursor population. **E** A subset of genes from the D50 THRB2^+^ early L/M cone Cluster 2 categorized into gene families such as transcription factors (21 genes), including THRB (bolded in red), cytokines/growth factors (11 genes), protein kinases (11 genes) and cell surface (CD) markers (4 genes), including CD166/ALCAM (bolded in red). **F** Bar chart of top 3 gene ontology (GO) biological processes for Cluster 2 based on the -log10(p-value). **G** Volcano plot of differentially expressed genes in D50 THRB2^+^ L/M cones vs. D100 THRB2^+^ L/M cones with the corresponding top three GO biological processes for each cell population shown on the left and right, respectively

Upregulated genes in each of the three FAC-sorted PR cell populations from the L/M-CRR line (D50 THRB2^+^, D100 THRB2^+^, and D100 NRL^+^) were identified by inputting read count data obtained via RSEM (RNA-seq by Expectation-Maximization) into edgeR (v3.16.5). Genes were designated as upregulated using a fold change cutoff *≥* 1.5 and a false discovery ratio (FDR) < 0.0001. A total of 1890 genes were found to be upregulated in one or more of the three positively sorted fluorescent cell populations compared to their negatively sorted counterparts. Genes upregulated in all three positively sorted groups included pan-PR markers such as *AIPL1*,* IMPG2*,* EYS*,* RCVRN*,* RD3*,* NEUROD1*,* CRX*, and *ABCA4* (Fig. 4A-C). As expected, there was a selective upregulation of cone genes in the D50 and D100 THRB2:tdTomato^+^ cell populations (e.g., *GNAT2*,* THRB*,* RXRG*,* ARR3*,* PDE6C*,* PDE6H*,* CNGB3*) (Fig. 4A, B), and a selective upregulation of rod genes in the D100 NRL:eGFP^+^ cell population (e.g., *NRL*,* NR2E3*,* GNAT1*,* PDE6B*,* PDE6G*,* FRMPD1* and *CNGB1*) (Fig. 4C). Of note, other known L/M cone-specific genes such as *VOPP1*, *MYH4*, and *LMO4* [58] were also selectively expressed in D100 THRB2:tdTomato^+^ cell populations (Fig. S5D), which further establishes THRB2:tdTomato^+^ cells as L/M cone-competent precursors.

K means clustering (K = 6) (Fig. 4D) of the 1890 upregulated genes revealed a total of 110 genes that were selectively upregulated in D50 THRB2^+^, D50 THRB2^–^, and D100 THRB2^+^ cells (Cluster 1), 294 genes in D100 THRB2^+^ cells only (Cluster 3), 423 genes in both D100 THRB2^+^ and D100 NRL^+^ cells (Cluster 4), 259 genes in D100 NRL^+^ cells only (Cluster 5), and 496 genes in D50 THRB2^+^, D100 THRB2^+^, and D100 NRL^+^ cells (Cluster 6). Of primary interest for the present study, however, was a unique cluster of 308 genes (Cluster 2) that were highly upregulated only in D50 THRB2^+^ L/M cone-competent precursor cells.

Gene Set Enrichment Analysis (GSEA) of Cluster 2 showed that it contained genes belonging to multiple families such as transcription factors (including THRB), cytokines/growth factors, protein kinases, and cluster of differentiation (CD) cell surface proteins, including CD166/ALCAM (Activated Leukocyte Cell Adhesion Molecule), a cell adhesion protein known to be involved in axon outgrowth [37, 59–63] (Fig. 4E). Gene ontology (GO) analysis of all genes belonging to Cluster 2 showed ‘Inorganic Cation Transmembrane Transport’, ‘Axon Guidance,’ and ‘Neuromuscular Process’ to be the top enriched biological processes (Fig. 4F). Of note, inorganic cation transmembrane transport is crucial for growth-cone protrusion, axonal pathfinding, and initial formation of synaptic contacts [64, 65]. *ALCAM* was also one of the genes listed in the top biological processes of cluster 2 (Axon Guidance) along with *SEMA6B*,* RET*,* NELL2*,* EPHA7*,* NRP2*,* UNC5B*,* NRXN1*,* SCN1B*, and *CDK5R1*. GO analysis also revealed distinct enriched biological processes for D50 vs. D100 THRB2^+^ L/M cone-competent precursor genes. More specifically, the top three GO biological processes for D50 (early) THRB2^+^ L/M cone-competent precursors were ‘Regulation of Cell Migration’, ‘Nervous System Development’, and ‘Axon Guidance’, while the top three for D100 (late) THRB2^+^ L/M cone-competent precursors were ‘Monoatomic Cation Transmembrane Transport’, ‘Action Potential’, and ‘Visual Perception’ (Fig. 4G and Tables S5, S6). These results are in concordance with our recent report showing that early PRs have greater axon terminal dynamics and migratory capacity compared to later PRs [11].

### Enrichment of early L/M cone-competent precursors from ROs using CD166/ALCAM-mediated MACS

**Fig. 5:**
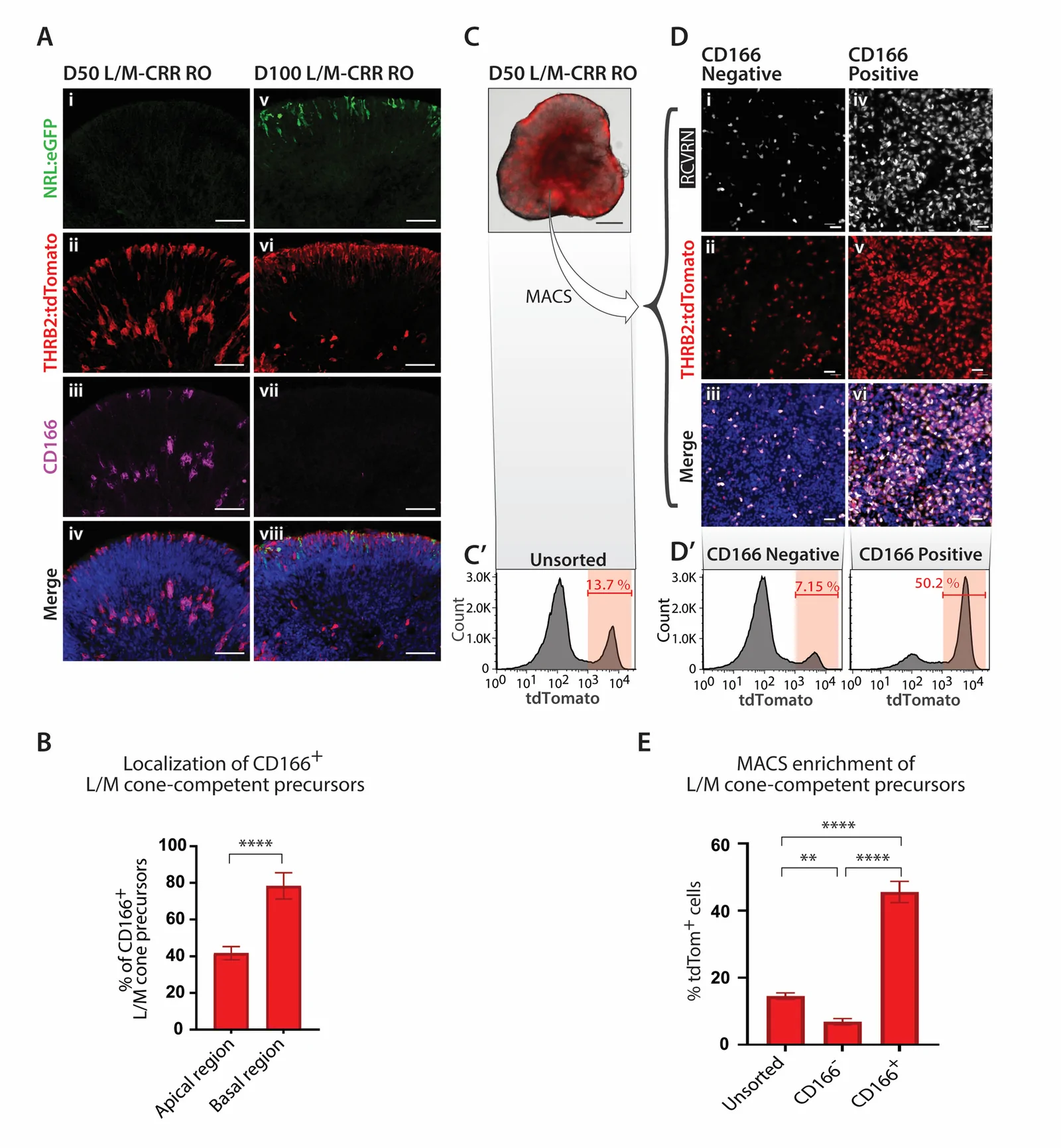
MACS enrichment of L/M cone-competent precursors using CD166/ALCAM. **A** Fluorescence microscopic images of D50 L/M-CRR ROs (i-iv) and D100 L/M-CRR ROs (v-viii) immunostained for eGFP (green) (i, v), tdTomato (red) (ii, vi), and CD166 (magenta) (iii, vii). Merged images including nuclei (DAPI, in blue) at D50 and D100 are shown in iv and viii, respectively. CD166/ALCAM is exclusively expressed in THRB2:tdTomato^+^ L/M cone-competent precursors at D50 and is absent in D100 ROs. **B** Bar graph showing the percentage of L/M cone-competent precursors expressing CD166/ALCAM in the apical and basal neuroepithelial regions of D50 L/M-CRR ROs (n = 5). Graphs are plotted as mean ± SD. The majority of CD166^+^ L/M cone-competent precursors are present in the basal half of the RO neuroepithelium. ****P < 0.0001; unpaired t test. **C**’ Fluorescent image of an intact D50 L/M-CRR-RO containing THRB2:tdTomato^+^ L/M cone-competent precursors. **C** Representative histogram showing the flow cytometric (FC) quantification of THRB2:tdTomato^+^ L/M cone-competent precursor cells from dissociated, unsorted D50 L/M-CRR ROs. **D** ICC images of plated cells from the negative (i-iii) and positive (iv-vi) sorted fractions following CD166-based magnetic-activated cell sorting (MACS) of dissociated D50 L/M-CRR ROs. Immunostaining is shown for tdTomato (red) (i, iv) and RCVRN (white) (ii, v), along with merged images that identify all cell nuclei (DAPI in blue) (iii, vi). **D**’ Representative histogram showing the FC quantifications of THRB2:tdTomato^+^ L/M cone-competent precursor cells in the negative and positive sorted fractions immediately after CD166 MACS. **E** Bar graph showing the percentage of THRB2:tdTomato^+^ L/M cone-competent precursors in the dissociated, unsorted L/M-CRR RO cell population versus the negative and positive fractions after CD166 MACS (> 20 D50-D60 ROs from *n* = 4 independent differentiations). Graphs are plotted as mean ± SD. ** *P* < 0.01, ****P < 0.0001 (One-Way ANOVA with Tukey analyses). Scale bars for panels **A** and **D** 50 μm; **C** 200 μm

We focused on CD166/ALCAM (Fig. 4E, bolded in red) in our efforts to enrich early L/M cone-competent precursors given (1) its highly differential gene expression in D50 THRB2:tdTomato^+^ L/M cone-competent precursors vs. D100 THRB2:tdTomato^+^ L/M cone-competent precursors and D100 NRL:eGFP^+^ rod precursors, (2) its known role in CNS axonogenesis, and (3) the availability of robust and specific primary antibodies. We first confirmed that CD166 protein is exclusively expressed in post-mitotic THRB2:tdTomato^+^ L/M cone-competent precursors at D50, and not in any cells in D100 ROs (Fig. 5A and Fig. S6). However, only 60.04 ± 3.58% of THRB2:tdTomato^+^ cells within individual ROs (*n* = 5) co-labeled with CD166 (Table S7). Interestingly, we also observed that CD166 was predominantly expressed in L/M cone-competent precursors located in the basal neuroepithelial region of D50 ROs compared to the apical region (Fig. 5A.iii and Fig. S6) [11, 55, 66, 67]. Prior evidence has shown that PRs undergo interkinetic migration between the apical and basal regions of the developing outer neuroblastic layer both in vivo and in ROs, with mature cones ultimately residing in the outermost apical region [11, 15, 68]. The predominant presence of CD166 in the basal region led us to hypothesize that this cell surface protein selectively marks migratory L/M cone-competent precursors. Image-based quantification in apical and basal regions of L/M-CRR ROs at D50 revealed 78.40 ± 7.18% of THRB2:tdTomato^+^ cells in the basal region expressed CD166 while only 41.68 ± 3.61% of THRB2:tdTomato^+^ cells in the apical region expressed CD166 (Fig. 5B, Table S8, Fig. S7). Together, these findings suggest that CD166 delineates a subset of early post-mitotic L/M cone-competent precursors that exist during a peri-migratory period.

We next sought to determine whether CD166/ALCAM-mediated magnetic activated cell sorting (MACS) could enrich for this transient population of dynamic L/M cone-competent precursors identified in D50 ROs. Flow cytometric (FC) quantification of dissociated and unsorted L/M-CRR ROs at D50 (Fig. 5C) showed that THRB2:tdTomato^+^ L/M cone-competent precursors comprise ~ 14% of the total RO cell population at D50 (representative flow plot in Fig. 5C’). Following MACS using a primary CD166 antibody, the positive sorted fraction was highly enriched with THRB2:tdTomato^+^/RCVRN^+^ L/M cone-competent precursors compared to the negative sorted fraction (Fig. 5D). FC quantification immediately after CD166 MACS of L/M-CRR ROs at D50 (representative flow plots in Fig. 5D’) showed that the CD166 positive fraction contained 45.55 ± 3.17% THRB2:tdTomato^+^ L/M cone-competent precursors while the negative fraction contained 6.86 ± 0.95% (*n* = 4 independent experiments using > 20 D50-D60 ROs per experiment) (Fig. 5E). Based on an average THRB2:tdTomato^+^ cell percentage of 14.53 ± 0.96 in unsorted L/M-CRR ROs (Fig. 5E), CD166 MACS was able to achieve > 3-fold enrichment of L/M cone-competent precursors.

### CD166/ALCAM identifies a transient population of early L/M cone-competent precursors with peak axon dynamics

**Fig. 6:**
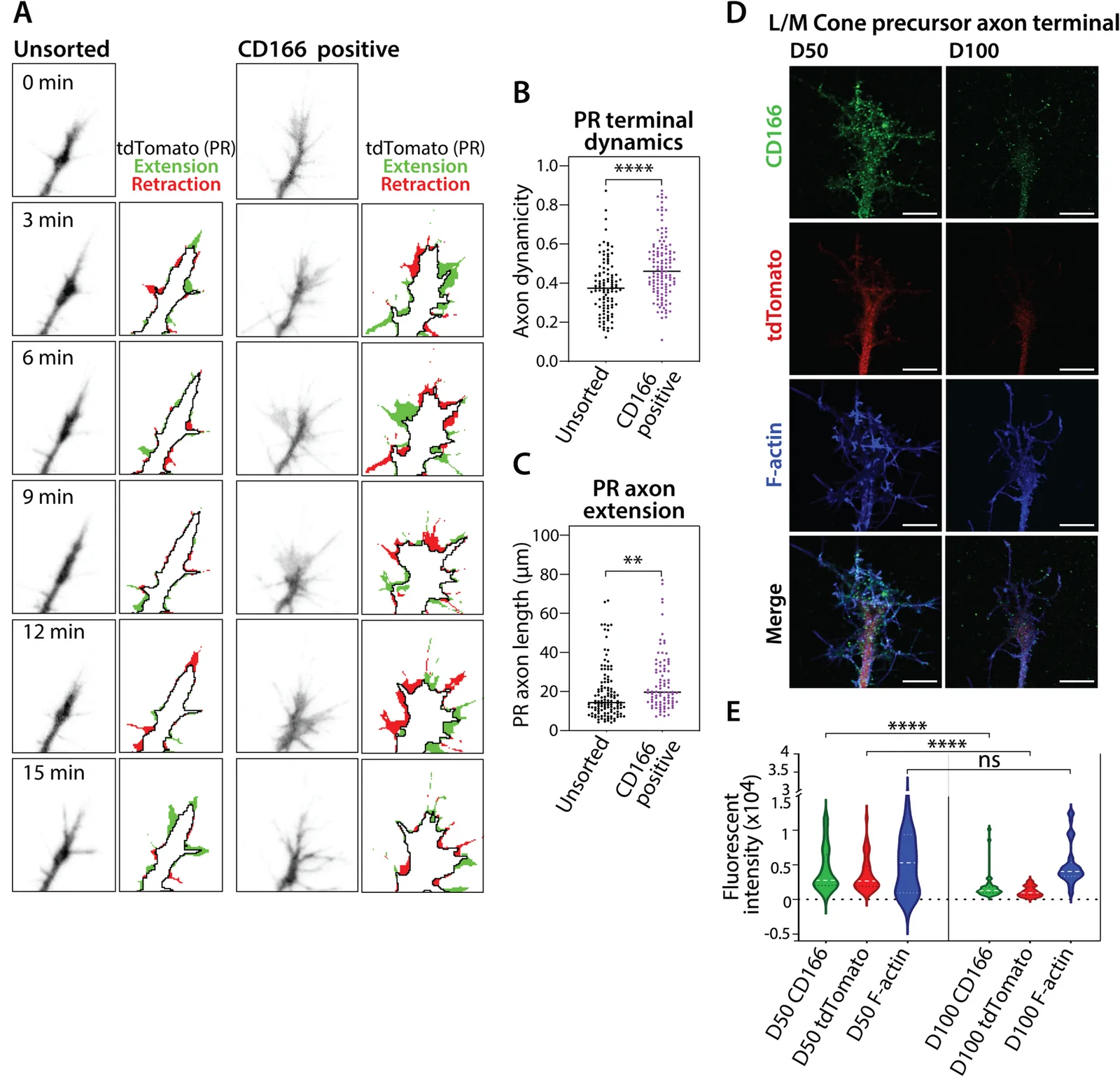
CD166 MACS enriches for RO-derived PRs with high axon dynamicity. **A** Time-lapse still images of representative axon terminals from dissociated D50 RO-PRs plated on poly-D-lysine and laminin-coated plastic [11] before (left panels) and after (right panels) CD166 MACS. For these experiments, we used ROs from a pan-PR hPSC reporter line (WA09 CRX^+/tdTomato^ [18]) that labels all PRs beginning at ~D28. Axon terminal expression of CRX: tdTomato is shown in grayscale over a 15 min period, with corresponding outlines and colored regions illustrating areas of axon extension (green) and retraction (red) between consecutive time points. **B** Axon dynamicity (*area of extension + area of retraction)/total area of terminal*) was measured from ≥ 100 CRX: tdTomato^+^ PR axon terminals in each group. The scatter plot shows relative axon dynamicity values (1 = high, 0 = low) for each axon terminal from unsorted (black data points) or CD166 MAC-sorted (purple data points) PRs. Horizontal lines indicate median values. ****P < 0.0001; unpaired t test. **C** Scatter plot showing CRX: tdTomato^+^ PR axon lengths (in microns) from unsorted (black data points) or CD166 MAC-sorted (purple data points) ROs 4 days after dissociation and plating (*n* > 80 CRX: tdTomato^+^ PR axon terminals for each group). Horizontal lines indicate median values. **P < 0.01; unpaired t test. **D** Representative ICC images of L/M cone-competent precursor axon terminals from D50 (left panels) or D100 (right panels) dissociated L/M-CRR ROs immunostained for CD166 (green), THRB2:tdTomato (red), and the synaptic cytoskeleton marker F-actin (blue). **E** Violin plot showing a comparison of fluorescence intensities of D50 (left) vs. D100 (right) L/M cone-competent precursor axon terminal images from panel **E** (*n* > 50 axon terminals for each group). ****P < 0.0001, ns = non-significant (One-Way ANOVA with Tukey analyses). Scale bars for panel **D** 10 μm

We previously showed that early (D40) RO-derived PR precursors display highly active axon terminals and a robust capacity to extend axons. Surprisingly, this early autonomous axonogenic potential was completely lost by D80 [11]. It therefore stands to reason that transplanted photoreceptors are more likely to successfully integrate and form connections to host neurons if they are harvested near D40. Given the relative paucity of post-mitotic PRs at this stage of RO differentiation, we sought to develop a cell surface marker enrichment strategy applicable to ROs derived from any hPSC line. Our current observation that CD166/ALCAM (a cell adhesion protein involved in axon growth [37, 59–63]) is transiently expressed in a subset of peri-migratory L/M cone-competent precursors at D50 led us to examine whether CD166 MACS-enriched PR precursors demonstrate greater axon dynamics than their unsorted counterparts. Of note, we chose to compare the axon dynamics of the positive CD166 MACS fraction with that of unsorted photoreceptors rather than the negative CD166 MACS fraction since most photoreceptor transplantation experiments currently utilize unsorted populations. To include all PR precursors in this analysis (i.e., CD166^+^ L/M cones, CD166^–^ L/M cones, S cones, and uncommitted PR precursors), we used our pan-PR reporter line (*CRX*^+/tdTomato^) [18], which is derived from the same WA09 hESC line used to create the L/M-CRR line and labels all RO PRs throughout RO differentiation starting at ~D28 [18]. The axon dynamicity ((*area of axon terminal extension + area of retraction)/total area of terminal*) of the D50 unsorted CRX: tdTomato^+^ PR population was then compared to that of the D50 CD166 magnetic-activated cell (MAC)-sorted CRX: tdTomato^+^ PR population, the latter being enriched for migratory L/M cone-competent precursors. Consistent with our hypothesis above, we found significantly higher axon dynamicity and extension in the sorted population (Fig. 6A-C, Fig. S8).

To further discern whether CD166/ALCAM may be important for L/M cone-competent precursor axon growth and dynamicity, we examined its expression in D50 vs. D100 THRB2:tdTomato^+^ axon terminals of dissociated L/M-CRR ROs. As expected, CD166 was prominently expressed in D50, but not D100, L/M cone-competent precursor axon terminals (Fig. 6D). To quantify our observations, we measured the fluorescence intensities (FI) corresponding to THRB2:tdTomato, CD166, and F-actin expression at D50 and D100 (Fig. 6E; *n* > 50 axon terminals for both time points). D50 L/M cone-competent precursor axon terminals possessed greater FIs for both THRB2:tdTomato and CD166 compared to corresponding D100 terminals, while the FI for F-actin, a general marker for axon terminals used as a control, was not significantly different. Collectively, these data suggest that CD166/ALCAM is not only a cell surface marker for a transient population of L/M cone-competent precursors with high axon dynamics, but may also play a direct role in cone axon outgrowth.

## Discussion

In this study, we report on the production of the first L/M cone-specific hPSC reporter line, which was combined with a rod-specific reporter [19] to also create the first dual cone-rod hPSC reporter (L/M-CRR) line. Our primary goal was to unambiguously identify hPSC-derived L/M cone-competent precursors at their earliest committed stage given recent findings that very early cones possess high axonogenic and migratory potential that rapidly declines during differentiation [11]. In addition, hPSC-cones, but not hPSC-rods, demonstrate intrinsic phototransduction activity akin to adult macaque retina, including the capacity to generate graded, repetitive, wavelength-specific light responses [10]. Lastly, only L/M cones, and not S cones, populate the human fovea and provide high acuity central vision, which is selectively lost in prevalent macular diseases such as age-related macular degeneration [69, 70].

We chose to link a fluorescent reporter to *THRB2* to identify early L/M cone-competent precursors since, in all species examined to date [23–25, 27, 29, 52–58], Thrb2 is the earliest marker of L/M cone competency, and in mice, Thrb2 knockout results in a complete absence of M cones [23, 25]. Consistent with this finding, simultaneous disruption of both THRB1 and THRB2 in hPSCs eliminated L/M cones in retinal organoids [28], presumably due to loss of THRB2 function, since THRB1 is widely expressed in the retina and numerous other tissues, whereas THRB2 expression is restricted to L/M cones. Importantly, hPSC reporter lines linked to cone genes other than THRB2 do not distinguish between S and L/M cones and, other than GNGT2 [22], demonstrate a later onset of expression [20, 21]. While other reported L/M cone specific genes, such as *VOPP1*, *MYH4* and *LMO4* [71], could be used to create L/M cone reporter lines, they are not expressed as early as *THRB2*.

To create the early L/M cone reporter portion of the L/M-CRR line, we inserted a *tdTomato-P2A* sequence between the endogenous *THRB2* TIS and the 5’ end of its first codon. This design was critical for three reasons. First, there is a lack of knowledge regarding critical THRB2 regulatory elements, which necessitates the use of the endogenous start site to faithfully recapitulate its expression. Second, THRB1 and THRB2 differ only at their N-termini due to the presence of alternative start sites [30]. As such, linking tdTomato to the 3’ end of the gene would have failed to distinguish between the two protein isoforms and negated the L/M cone-specificity of the reporter line. Third, by preserving the normal biallelic expression levels of THRB1 and THRB2, we avoided potential confounding effects that could arise due to haploinsufficiency of either isoform. Of note, this last consideration is not applicable to the NRL-GFP rod reporter portion of the L/M-CRR line since humans who are heterozygous for mutant *NRL* have a normal retinal phenotype [72].

Immunocytochemical characterization of ROs produced by the L/M-CRR line showed initial expression of THRB2:tdTomato in a subset of cells corresponding to the predicted birthdate and spatial distribution of cone precursors [11, 56, 66, 67], 73– [55]. THRB2:tdTomato expression remained strong until late stage 2 [8], when it began to diminish in agreement with the reported in vivo temporal expression profile of Thrb2 [24, 25]. Interestingly, THRB2:tdTomato^+^ cells were initially found in both the basal and apical regions of the developing RO neuroepithelium but became increasingly restricted to the apical surface where cone photoreceptors are known ultimately to reside [8]. This spatiotemporal pattern is consistent with the postmitotic movement of newborn cone photoreceptor precursors and likely reflects greater migratory potential of early THRB2:tdTomato^+^ cells.

Although the localization of THRB2:tdTomato^+^ cells in ROs was identical to that of early cones, their definitive assignment as L/M cone-competent precursors required a series of investigations based on a predetermined set of inclusion and exclusion criteria. First, we confirmed that early THRB2:tdTomato^+^ cells belonged to the cone lineage by demonstrating strict co-localization of tdTomato with the general cone marker ARR3. Next, we examined later stage ROs at a time point shortly before the loss of tdTomato signal, when S-Opsin expression is robust and L/M-Opsin is first detectable by immunocytochemistry [8]. THRB2:tdTomato^+^ cells in these older ROs co-localized only with L/M-Opsin and never with S-Opsin. Lastly, since NRL:eGFP^+^ rod precursors are prevalent at intermediate to late stages of differentiation, we could exclude the THRB2:tdTomato^+^ cell population from the developing rod population. Together, these results definitively assign all THRB2:tdTomato^+^ cells present in our L/M-CRR ROs to the L/M cone lineage. Importantly, like most RO differentiation protocols, our culture medium includes triiodothyronine (T3), the ligand for all THRB isoforms, beginning shortly after RO isolation. Thus, culture conditions are generally conducive to L/M cone formation.

Once it was determined that the THRB2:tdTomato signal faithfully marked postmitotic L/M cone-competent precursors, we subjected differentiating L/M-CRR ROs to FACS followed by bulk RNAseq to define their molecular signature over time. In particular, we sought to delineate the gene expression profile of very early L/M cone-competent precursors, which are known to have high migratory and axon outgrowth potentials that are largely lost by D80 [11]. To do so, we first looked for genes in D50 L/M cone-competent precursors that were absent in all other D50 RO cell types. We then refined this list by excluding genes found in D100 rod precursors or in older, D100 L/M cone-competent precursors. Analysis of the resulting very early L/M cone-competent precursor-specific gene cluster showed inorganic cation transmembrane transport (crucial for neuronal migration and synapse formation [64, 65]) and axon guidance to be the top enriched biological processes, and further identified multiple transcription factors, cytokines, and protein kinases that are uniquely enriched in this population, most of which were not previously known to be involved in retinal development. Such information can provide new insights into potential mechanisms of human L/M cone genesis and assist efforts to augment L/M cone production for the study and treatment of macular disorders. Of note, a unique gene cluster was also discovered for very early (D100) rod precursors, which is of similar interest and utility for rod-based degenerative diseases such as retinitis pigmentosa.

While expression of THRB2:tdTomato facilitates rapid and efficient isolation of early L/M cone-competent precursors in the L/M-CRR line, it would be ideal to enrich for this population in any hPSC line. To achieve this goal, we identified a cell surface marker, CD166/ALCAM, that (1) is highly expressed in our D50 L/M cone-competent precursor dataset and (2) has a robust, commercially available primary antibody. Of note, while three other cell surface marker genes were selectively upregulated in D50 L/M cone-competent precursors (*CD52*,* ENTPD1/CD39* and *LEPR/CD295*), they either had low gene count values or their corresponding proteins could not be definitively detected in ROs by ICC using available primary antibodies. In contrast, immunocytochemistry for CD166/ALCAM was robust and confirmed that it is expressed exclusively in a subset of THRB2:tdTomato^+^ L/M cone-competent precursors. Interestingly, ALCAM is a cell adhesion protein that is involved in neuronal migration as well as axon growth and guidance [37, 59–63]. It is therefore likely that ALCAM has a functional role in bestowing transient pro-integration properties to very early L/M cone-competent precursors. Indeed, ALCAM is one of the expressed genes listed in the ‘Axon Guidance’ biological process category, along with *SEMA6B*, *RET*, *NELL2*, *EPHA7*, *NRP2*, *UNC5B*, *NRXN1*,*SCN1B*, and *CDK5R1*. Furthermore, CD166/ALCAM preferentially labelled L/M cone-competent precursors located within the basal migratory region of the developing RO neuroepithelium.

Evidence of CD166/ALCAM expression in developing vertebrate retina has been reported in fish, chicks and mice, but thus far only in retinal ganglion cells (RGCs), where it is localized along extending axons [37, 63, 75–82]. However, in addition to defects in RGC axon fasciculation, mice lacking CD166/Alcam also demonstrate retinal dysplasia and photoreceptor mis-localization, consistent with abnormal photoreceptor migration [75]. While these findings suggest a role for CD166/ALCAM in the early development of both RGCs and photoreceptors in vivo, we did not detect CD166/ALCAM expression in the negatively sorted D50 THRB2:tdTomato retinal cell population (which contains RGCs) or in fluorescently labeled D36 RGCs differentiated from a E4-H7 BRN3B: tdTomato RGC reporter line [40] (Fig. S9). Thus, our current findings support a more restricted role for CD166/ALCAM in L/M cone-competent precursors at very early time points during RO differentiation (i.e., stage 1 [8]).

Using a primary antibody directed against CD166/ALCAM, we achieved an ~ 3-fold MACS enrichment of THRB2:tdTomato^+^ L/M cone-competent precursors from D50 ROs. However, given that CD166/ALCAM is expressed in only ~ 60% of D50 THRB2:tdTomato^+^ cells, this result corresponds to an ~ 5-fold enrichment of the CD166/ALCAM^+^ L/M cone-competent precursor population. Finally, to test whether a subpopulation of CD166-sorted PRs possesses an enhanced capacity for axon outgrowth relative to all other coexisting PRs, we employed a previously described assay that quantifies key parameters of axonogenesis [11]. D50 PRs had a significantly higher average axon length and growth cone dynamicity following CD166/ALCAM-mediated MACS compared to unsorted D50 PRs from the same RO cultures. High resolution microscopy confirmed that CD166/ALCAM is prominently expressed in axon terminals of D50 L/M cone-competent precursors, consistent with its postulated role in promoting axon outgrowth. Interestingly, although CD166/ALCAM was not found in older intact ROs, L/M cone-competent precursors dissociated from D100 ROs and plated on a laminin substrate showed weak CD166/ALCAM expression at their axon terminals. This finding implies a capacity for older L/M cone-competent precursors to re-acquire limited axonogenic potential.

## Conclusions

Using a novel L/M-CRR line, we identified a transient population of early L/M cone-competent precursors that can be identified and enriched from stage 1 ROs via CD166/ALCAM expression. CD166/ALCAM^+^ L/M cone-competent precursors are predominantly localized to migrating postmitotic cells that display superior axon dynamics, properties that are highly desirable for L/M cone replacement strategies. CD166/ALCAM’s known role as an adhesion protein involved in CNS axon growth and guidance further suggests that it is not only a fortuitous cell surface marker, but also functionally involved in the proper positioning and wiring of L/M cones during development. Given that L/M cones are the sole PR subtype responsible for central (i.e., macular) vision, we anticipate that these findings will facilitate the development of advanced cell therapeutics tailored for macular diseases.

## Supplementary Information

Below is the link to the electronic supplementary material.

Data S1

Data S2

Data S3

Supplementary Figures and Tables

## Data Availability

The RNAseq data (both raw data and processed data) supporting the results reported in this manuscript are archived in GEO public repository (Accession number GSE310055). Quick link: [https://www.ncbi.nlm.nih.gov/geo/query/acc.cgi?&acc=GSE310055](https:/www.ncbi.nlm.nih.gov/geo/query/acc.cgi?&acc=GSE310055). All other data supporting the findings of this study are available in the main text or the supplementary materials. The L/M-CRR hPSC line will be made available upon request following completion of a material transfer agreement (MTA).

## Abbreviations

L/M cone: Long/medium wavelength cone
CRR: Cone/rod reporter
CRISPR: Clustered regularly interspaced short palindromic repeats
hPSC: Human pluripotent stem cell
RO: Retinal organoid
D: Days
FACS: Fluorescence-activated cell sorting
MACS: Magnetic-activated cell sorting
PR: Photoreceptor
THRB2: Thyroid hormone receptor beta 2
NRL: Neural retina-specific leucine zipper
eGFP: Enhanced green fluorescent protein
ALCAM: Activated leukocyte cell adhesion molecule
hESCM: Human embryonic stem cell medium
NEAA: Non essential amino acids
NIM: Neural induction medium
RDM: Retinal differentiation medium
FC: Flow cytometry
ICC: Immunocytochemistry
RT: Room temperature
TPM: Transcripts per million
iMFI: Integrated median fluorescence intensity
