# Supplementary material for: Identification and enrichment of human retinal organoid-derived red/green cone-competent precursors with enhanced axon dynamics
Source: Stem Cell Res Ther. 2026 Jul 4;17:336. doi: 10.1186/s13287-026-05128-9 (PMC13613627; doi:10.1186/s13287-026-05128-9)
Supplement: Supplementary file 1 [file 13287_2026_5128_MOESM1_ESM.docx]

**Data S2.**

Confirmed Sanger sequences of the top 10 predicted off-target sites in L/M-CRR clones 24 and 38 showing the absence of indels.

**Off-target amplicons for sgRNA1.** 10 off-target sites for sgRNA1 were sequenced for clones 24 and 38 of the L/M-CRR line. No indels were detected in either clone. Off-target sequences are noted in red and primer binding sequences are underlined.

**L/M-CRR clone 24** sgRNA1 OT1: AC022116.5: 153766 to 154514 Homo sapiens chromosome 5.

GTGGTTATTGAAGGAGTCCTCCATGGGATATTTTACCCTCATCTACAGCCCAGGTAGAAATCCTACATGGCTAAAAGAAGCAGAAGCAAGTTTCGAAGTCACAGTCAAGGAAATCAATACCTACCAATTTAACCTAAACGCTATGATATATAACAGCTCTAGCTAGTGGTAAAGTGCACAGTCCCAGCTTAATTCAGGGCAGGGACATTTCCATTAGAATGGTGCTCTTAAAAATAGAAACTGAACCGGGGCGGTGGTCAGGCTAAGGCCAAGTGTTTAAGAAGTAGAGTGTAGCTGCCAGCGTAGAAACCCATGAAAAGGAGGCCACAGGAGATTCCTGGGAGCACTGGGTGTAGCAAAACAAAGCCACTCTCTGCTTCAGTCGCACCATTTGCTAATTGAAAATCATATCCTGAATCATACTGAGACTGATCAACTTTGGTAGCTTTTTTGTTCAGATCTTATGACACACTACTCTTCTCACCGTGAGATTTTCTCAGCCAGTGATAGTACATTCTGAAATGCTGGCACCAGGAGACGGCCACAGACACACACTGCTAAATGTGAAGATGGAACTAAACTGGAAATTAAATTATACTGACAATATTATGGCATTTTTAAGATCATGGCATTTTAATTTACATTAGAGTGGAGTTGCATCATACTCAGGGGTTAGCTTCCAAGGTCAGTACATAGGTAAAATGGGCTATTAGGATGATCCTTGAAAGCCCTTTAGAAGGGTGCCATGT

**L/M-CRR clone 38** gRNA1 OT1: AC022116.5: 153766 to 154514 Homo sapiens chromosome 5.

GTGGTTATTGAAGGAGTCCTCCATGGGATATTTTACCCTCATCTACAGCCCAGGTAGAAATCCTACATGGCTAAAAGAAGCAGAAGCAAGTTTCGAAGTCACAGTCAAGGAAATCAATACCTACCAATTTAACCTAAACGCTATGATATATAACAGCTCTAGCTAGTGGTAAAGTGCACAGTCCCAGCTTAATTCAGGGCAGGGACATTTCCATTAGAATGGTGCTCTTAAAAATAGAAACTGAACCGGGGCGGTGGTCAGGCTAAGGCCAAGTGTTTAAGAAGTAGAGTGTAGCTGCCAGCGTAGAAACCCATGAAAAGGAGGCCACAGGAGATTCCTGGGAGCACTGGGTGTAGCAAAACAAAGCCACTCTCTGCTTCAGTCGCACCATTTGCTAATTGAAAATCATATCCTGAATCATACTGAGACTGATCAACTTTGGTAGCTTTTTTGTTCAGATCTTATGACACACTACTCTTCTCACCGTGAGATTTTCTCAGCCAGTGATAGTACATTCTGAAATGCTGGCACCAGGAGACGGCCACAGACACACACTGCTAAATGTGAAGATGGAACTAAACTGGAAATTAAATTATACTGACAATATTATGGCATTTTTAAGATCATGGCATTTTAATTTACATTAGAGTGGAGTTGCATCATACTCAGGGGTTAGCTTCCAAGGTCAGTACATAGGTAAAATGGGCTATTAGGATGATCCTTGAAAGCCCTTTAGAAGGGTGCCATGT

**L/M-CRR clone 24** sgRNA1 OT2: AL354693.6: 67597 to 68316 Homo sapiens chromosome 1.

CTCACTGCAACCTTCACTTCCCAGGTTCAAGAGATTCTCCTGTCTCAGCCTCCCGAGTAGCTGGGATTACAGGCCCCTGCCATTACGCCCAGCTACTTTTTGTATTTTTAGTAGAGATGGGTTTCACCGTGTTGGTTAGGCTGGTTTGAACTCCTGAGCTCAGGCGATCTGCCTGCTTCAGCCTCCCAAAATGCTGGGATTACAGCCGTGAGCCACCGCCCCCAGCCGGTATTTTTTCAAATCAAAGAAAAAATAATAGAGTAAATCATCCAAAACTTTAGATGGTATTTAGACTCAGTAAACTTTTCATATATGACAGATGAAGCCAAATGGTCTTTCTGTGCAGTCAGCTAGCACACAATTGTGCACCCGAGGAAAATTAGAGACTGAACCGGGGTGTCTGTGGATGCATTTCCTCAGCATTCAGCCTTCCTTTTGCCCGTGTTCTAGCATTACTTCTGTCCTACAGCCTGGGATTTGTGAATGAAATAGACAGGTGCAAAAACTCCCTGCCTGTCTGTAATATCCATAGCCCCGTGCTCTACTTGTATTTGCATGTACAAACCATAATCTCCTGTAAAATACTCTGTGATATTTCTGAATAATAATAAACTCTACATCCTACACAAAGGCAAAACCCCTGTATCTTTCATCTTTGAAACCATAGCAAAGGTATGAAATTACACCTGAGCATGCCTGGCCTCAAAGTCCTGGAACGGT

**L/M-CRR clone 38** sgRNA1 OT2: AL354693.6: 67597 to 68316 Homo sapiens chromosome 1.

CTCACTGCAACCTTCACTTCCCAGGTTCAAGAGATTCTCCTGTCTCAGCCTCCCGAGTAGCTGGGATTACAGGCCCCTGCCATTACGCCCAGCTACTTTTTGTATTTTTAGTAGAGATGGGTTTCACCGTGTTGGTTAGGCTGGTTTGAACTCCTGAGCTCAGGCGATCTGCCTGCTTCAGCCTCCCAAAATGCTGGGATTACAGCCGTGAGCCACCGCCCCCAGCCGGTATTTTTTCAAATCAAAGAAAAAATAATAGAGTAAATCATCCAAAACTTTAGATGGTATTTAGACTCAGTAAACTTTTCATATATGACAGATGAAGCCAAATGGTCTTTCTGTGCAGTCAGCTAGCACACAATTGTGCACCCGAGGAAAATTAGAGACTGAACCGGGGTGTCTGTGGATGCATTTCCTCAGCATTCAGCCTTCCTTTTGCCCGTGTTCTAGCATTACTTCTGTCCTACAGCCTGGGATTTGTGAATGAAATAGACAGGTGCAAAAACTCCCTGCCTGTCTGTAATATCCATAGCCCCGTGCTCTACTTGTATTTGCATGTACAAACCATAATCTCCTGTAAAATACTCTGTGATATTTCTGAATAATAATAAACTCTACATCCTACACAAAGGCAAAACCCCTGTATCTTTCATCTTTGAAACCATAGCAAAGGTATGAAATTACACCTGAGCATGCCTGGCCTCAAAGTCCTGGAACGGT

**L/M-CRR clone 24** sgRNA1 OT3: AC026248.7: 63264 to 64071 Homo sapiens chromosome 8.

GGGTAGACACCAAGGAGCGCAATTATTGGATCACATGGTGAGACTATGTTTAGCTCATTAGGAAACTGCCATAGCTATATGTTTTTTGCATTTGTTCAGAGATTTTTCGTTACAATGTCACTAACATGATTAATCCTCAGAATAAACCTTTGGGTTGGGGGTTATTAGCCTTGCCTTCTGGCTAATGTTATTAAATGTTATTGCTGAAAGAAACCCTGAAGGTTCCTAAGCCTCTAAGCTTTCTGAGTGTGAGATCCACATCCCCTATCACTGCTCTTCTATGGCTTCCATGGCACCGTGAGTATAATAGTTGCTGAATTAGTGTCCACTACACACTGAATACCAATAATGGAAAAACCACTTCACTGTACAGATGAAAACACAAAGGTCCCAAAACGAGAAATGAGATACTTCTGGCCAAACGGCATTTTAACTAGTAACAGAACCAAGGCTAAAACTAAACTATTGTAGATCCGTAGACCTGTATTTTGAGCCACCAGTCCTTGACTCGCTACATTCAAGCAAGACAAGCTGGAAGATTCTTAAATCTGTAATTAGTATTGTATTGAACACCCCCCCTCACTTTCAGGCCTACCCACTACTGACTGCCATTACCAGATAGCCCAAAGACGCCCAAAATGGTGTTCTAATCTGAGTACAAGGCCACTATAGCTACAAAGTCTCATAGGGTAATGAGTGATAATAGCAGTGACAACAATAATAAAAATAGCCAAGAATTATTGATTGCTTAGTATTGTCAGGGTCTTTGCATATATTATTTCATTTTACACTCATAGCTGCCCCTTGG

**L/M-CRR clone 38** sgRNA1 OT3: AC026248.7: 63264 to 64071 Homo sapiens chromosome 8.

GGGTAGACACCAAGGAGCGCAATTATTGGATCACATGGTGAGACTATGTTTAGCTCATTAGGAAACTGCCATAGCTATATGTTTTTTGCATTTGTTCAGAGATTTTTCGTTACAATGTCACTAACATGATTAATCCTCAGAATAAACCTTTGGGTTGGGGGTTATTAGCCTTGCCTTCTGGCTAATGTTATTAAATGTTATTGCTGAAAGAAACCCTGAAGGTTCCTAAGCCTCTAAGCTTTCTGAGTGTGAGATCCACATCCCCTATCACTGCTCTTCTATGGCTTCCATGGCACCGTGAGTATAATAGTTGCTGAATTAGTGTCCACTACACACTGAATACCAATAATGGAAAAACCACTTCACTGTACAGATGAAAACACAAAGGTCCCAAAACGAGAAATGAGATACTTCTGGCCAAACGGCATTTTAACTAGTAACAGAACCAAGGCTAAAACTAAACTATTGTAGATCCGTAGACCTGTATTTTGAGCCACCAGTCCTTGACTCGCTACATTCAAGCAAGACAAGCTGGAAGATTCTTAAATCTGTAATTAGTATTGTATTGAACACCCCCCCTCACTTTCAGGCCTACCCACTACTGACTGCCATTACCAGATAGCCCAAAGACGCCCAAAATGGTGTTCTAATCTGAGTACAAGGCCACTATAGCTACAAAGTCTCATAGGGTAATGAGTGATAATAGCAGTGACAACAATAATAAAAATAGCCAAGAATTATTGATTGCTTAGTATTGTCAGGGTCTTTGCATATATTATTTCATTTTACACTCATAGCTGCCCCTTGG

**L/M-CRR clone 24** sgRNA1 OT4: AC005726.1: 48119 to 48734 Homo sapiens chromosome 17.

TGTCACCCAGGAGTTCAATGGCACAATCTTGGCTCACTGCAACCTTGGCCTCCAGGGTTCAAGCAGTTCTCCTGCTTCAGCCTTCTGAGTAGCTGGGACTACAGGTGTGCAACACCACACCCAGCTAATTTTTGTATTTTTTTAGTAGACACAAGGTTTCACCATGTTGGCCACGCTGGTCTCGAACTCTTGACCTCAACTGATCTGCCCACCTTGGCCTCCCAAATAACCTTAAATCTTAAGGTTGATGCATGCCTCTTAATCTCTTGGTTTGCCCATCAGAAACTGAATCATGGGCTCACTAGGAGAAATTGTCAGTTTAAAAATCCTATGCTTTTCAAAGAGGTTTTATTTTGGATAACTGTTGCAATTATAGACCTAGAGAAGACATTTAACCCACCCATGTTTCAGTTTCTATTTTCTTCCTCACTAGATTATTTTATTCACTTTTCAATTTTTAAAAATTAGATTTATTGCAGACTGGCCTCAAGCTCCCAGACTATATTTTTTAGCTGCATTATTTAGGGGTTGAAATGCCAGCTTTTGAGACTTTTGAAAAATTCACAGGCTGGGTGTGGAGGCTCATGCCTGTGAAGGTTGCAGTGAGCTGAGATCG

**L/M-CRR clone 38** sgRNA1 OT4: AC005726.1: 48119 to 48734 Homo sapiens chromosome 17.

TGTCACCCAGGAGTTCAATGGCACAATCTTGGCTCACTGCAACCTTGGCCTCCAGGGTTCAAGCAGTTCTCCTGCTTCAGCCTTCTGAGTAGCTGGGACTACAGGTGTGCAACACCACACCCAGCTAATTTTTGTATTTTTTTAGTAGACACAAGGTTTCACCATGTTGGCCACGCTGGTCTCGAACTCTTGACCTCAACTGATCTGCCCACCTTGGCCTCCCAAATAACCTTAAATCTTAAGGTTGATGCATGCCTCTTAATCTCTTGGTTTGCCCATCAGAAACTGAATCATGGGCTCACTAGGAGAAATTGTCAGTTTAAAAATCCTATGCTTTTCAAAGAGGTTTTATTTTGGATAACTGTTGCAATTATAGACCTAGAGAAGACATTTAACCCACCCATGTTTCAGTTTCTATTTTCTTCCTCACTAGATTATTTTATTCACTTTTCAATTTTTAAAAATTAGATTTATTGCAGACTGGCCTCAAGCTCCCAGACTATATTTTTTAGCTGCATTATTTAGGGGTTGAAATGCCAGCTTTTGAGACTTTTGAAAAATTCACAGGCTGGGTGTGGAGGCTCATGCCTGTGAAGGTTGCAGTGAGCTGAGATCG

**L/M-CRR clone 24** sgRNA1 OT5: AL356377.12: 36041 to 36822 Homo sapiens chromosome 1.

GGGTGTGATTAGCTGTGTGTCGAGTGCTATTGAGATTTTAAGTAAGATAAAAACTGAGAATAACCACTGGATTTAGCAACATGGAAATTAAAGGTGACCTTGACCAGTACAGTTTCAGCAGAGTAGTGAGGGGTCAAAGCCTAATTGGGGTAGATTCAGGTGAAAACAGAAGAAGAGAAATTACATTTTTAAATTTTAAGATATTTTGCAGTAATGGGGAGGAGGAAATTGGGGTCAAGAGAGGTTTTCTTTCTTCCTTTTAGGATGGGAGAAATAACAGACTATTTGCATGCTTTCAGAAATATTCAGAAGAGAGAGGAATATTGATGATACAGGAGAGGAGAAGAATTGCTAGAGTGATGTCTTTGAGCATGTTTTGGGGATATGGTAGCATAAGCCATCTGGCAGCAGTGATGCAGACACCGGTCAGATACTGGGAAGGCCACGTAAGCAAGTGACTGAACCAAGGATGTAGAATGAAGGCTGGGATCAGGGCTGTAGGCTGAGGTCCGAGCCCAAGTCAGGAACGTGCAGGCCCAGCACAAGTAAGCAGGAGGCCCCGTCAGAGGAGTAACCAGAGACTCTGTCCTGTAGGTTACTGAGTCAGGATTCGCCCGAGAAACAGAACATATAGGATACAAGAGGAGATTTATTGTGGGAATTGACTCACACATTTAGAGGCTGAGAAGTCCCAGAGTATGCCATCTTGCAAGCTGTAAAATGAGGGAAGCCAGCAGAATAGCTCAGTCTAAGTCTGAAGGCCTGAGAAACAGAGCAGCTGA

**L/M-CRR clone 38** sgRNA1 OT5: AL356377.12: 36041 to 36822 Homo sapiens chromosome 1.

GGGTGTGATTAGCTGTGTGTCGAGTGCTATTGAGATTTTAAGTAAGATAAAAACTGAGAATAACCACTGGATTTAGCAACATGGAAATTAAAGGTGACCTTGACCAGTACAGTTTCAGCAGAGTAGTGAGGGGTCAAAGCCTAATTGGGGTAGATTCAGGTGAAAACAGAAGAAGAGAAATTACATTTTTAAATTTTAAGATATTTTGCAGTAATGGGGAGGAGGAAATTGGGGTCAAGAGAGGTTTTCTTTCTTCCTTTTAGGATGGGAGAAATAACAGACTATTTGCATGCTTTCAGAAATATTCAGAAGAGAGAGGAATATTGATGATACAGGAGAGGAGAAGAATTGCTAGAGTGATGTCTTTGAGCATGTTTTGGGGATATGGTAGCATAAGCCATCTGGCAGCAGTGATGCAGACACCGGTCAGATACTGGGAAGGCCACGTAAGCAAGTGACTGAACCAAGGATGTAGAATGAAGGCTGGGATCAGGGCTGTAGGCTGAGGTCCGAGCCCAAGTCAGGAACGTGCAGGCCCAGCACAAGTAAGCAGGAGGCCCCGTCAGAGGAGTAACCAGAGACTCTGTCCTGTAGGTTACTGAGTCAGGATTCGCCCGAGAAACAGAACATATAGGATACAAGAGGAGATTTATTGTGGGAATTGACTCACACATTTAGAGGCTGAGAAGTCCCAGAGTATGCCATCTTGCAAGCTGTAAAATGAGGGAAGCCAGCAGAATAGCTCAGTCTAAGTCTGAAGGCCTGAGAAACAGAGCAGCTGA

**L/M-CRR clone 24** sgRNA1 OT6: AC011356.6: 30025 to 30914 Homo sapiens chromosome 5.

ACATCCGAATCCATCAGTGGCAATAAAAGAAAACCTGTTTTAATGAGATGCAACACATGTCTTTTGTTTGTGAAGGGCCTTACATCACAAGGTGTCCATTCTATGATTTGTTTCTTTAATTCTCTACACAAACCTGCCTTTTAATCAAACAGATTCAAAACACCAGGATCCTGCCTATTCCTGCACCTGACCTCCTTCCTCCCTTTGCTTTGTAAAACAGCCCCATTTCATTGCCTGCTGGGGGTCCGCTCTGAGTTGATATACATACAGTGGAACCTTTGCCCTGGTTTAATTTTTAGTTTCCACCCTTAATATTTATTAATATTACTATTATAAAACATCCCTTTTAAATAATCTGTTCACAATTAATGAACAGGCTATAAAAATATAGGTTCTGAGGCAAATAGCCCAGTGAAATACTTCGCTTGAAGGCTTTAGCCTAATTCATTGTTAGGTTTTCTTCCAAGAAAAAAATCAAATGCGCATCAGTGTTTGTTTGGTTCTGACACATAGAGTAGGTAAAACTTACCTGCCAAATGTCAACAGAATGTCTTCTTGGTCTTCCCAGGGATCTCAAGAAAATTGTTCAGCCTAGCTCTAAACTGGACTATACTTCACCCAAACAAAGGCAAATTGTGGATGGTTTAAAAACAAAACAACAAACTAAACTAGTAGAATAAGTGTATTATACAGAAGTGGGTGCCTCAAAGGTAGTGCTAAAGTGTCATATCTCCCAAATGTCTGTGCTAAAACTCTGAGTAATGGCAACCATTGCAGAAAAGAGAAGCTGTATTTCCTAGAAATTTTCTTGGGCATACTGGCTCCTGAGGATTTATCCCAAGGTAATAATGCCAGGTATTAATGAAAAATGTCTGTGTCATCCTGAAGGG

**L/M-CRR clone 38** sgRNA1 OT6: AC011356.6: 30025 to 30914 Homo sapiens chromosome 5.

ACATCCGAATCCATCAGTGGCAATAAAAGAAAACCTGTTTTAATGAGATGCAACACATGTCTTTTGTTTGTGAAGGGCCTTACATCACAAGGTGTCCATTCTATGATTTGTTTCTTTAATTCTCTACACAAACCTGCCTTTTAATCAAACAGATTCAAAACACCAGGATCCTGCCTATTCCTGCACCTGACCTCCTTCCTCCCTTTGCTTTGTAAAACAGCCCCATTTCATTGCCTGCTGGGGGTCCGCTCTGAGTTGATATACATACAGTGGAACCTTTGCCCTGGTTTAATTTTTAGTTTCCACCCTTAATATTTATTAATATTACTATTATAAAACATCCCTTTTAAATAATCTGTTCACAATTAATGAACAGGCTATAAAAATATAGGTTCTGAGGCAAATAGCCCAGTGAAATACTTCGCTTGAAGGCTTTAGCCTAATTCATTGTTAGGTTTTCTTCCAAGAAAAAAATCAAATGCGCATCAGTGTTTGTTTGGTTCTGACACATAGAGTAGGTAAAACTTACCTGCCAAATGTCAACAGAATGTCTTCTTGGTCTTCCCAGGGATCTCAAGAAAATTGTTCAGCCTAGCTCTAAACTGGACTATACTTCACCCAAACAAAGGCAAATTGTGGATGGTTTAAAAACAAAACAACAAACTAAACTAGTAGAATAAGTGTATTATACAGAAGTGGGTGCCTCAAAGGTAGTGCTAAAGTGTCATATCTCCCAAATGTCTGTGCTAAAACTCTGAGTAATGGCAACCATTGCAGAAAAGAGAAGCTGTATTTCCTAGAAATTTTCTTGGGCATACTGGCTCCTGAGGATTTATCCCAAGGTAATAATGCCAGGTATTAATGAAAAATGTCTGTGTCATCCTGAAGGG

**L/M-CRR clone 24** sgRNA1 OT7: AC087683.5: 161702 to 162399 Homo sapiens chromosome 18.

CAGCTGTTCAGGAGGCTAAGGTGGGAGGATTGCTGGAGCCCAGGAGGTTGAGGCTGCAGTAAGCTGTGATTGCACCACTGCACTCCAGCCTGGGCAACAGAGCAAAACTTCGATTCAGGAAAAAGGAAAGGAAGGGAAGGGAGGGGAAGGGAAGGGAAGGGAAGGGAAGGGGGAAGGAAGGAAGGAAGGGAAGGAAAGAAAGGAAGGAAGGAAGAAATGGGGCACAATCAACACATTTCTAAGCCATCATTAAAACTGGTTACTAGAATAATTAAAATAGAAATTGAAACAAGGTGGCAAAAGAAGCACAAAGCCAAAACTGCAACTCCAAGGGAAAATTAAATATGGGATCATATAATAATCATTGGAAGAGCCTACCACACCAAGAAAACACCTATTTCTGTAAGTTATTTTTCTGCAGACAGGCCACCGTGGACTCTTGCAATGCAAATTGTATCCCATAATTATGAGCGTATGTTATATTCATGATTCATGAAAATCACATGGGAAGGAAAAAATCATTCCTCTCCTTTCAAAATAAGATGCTTAAGGCTTCCTTTTCTCTTATAGAGATGGCCTTGTTAATTGAATTCTGATACTCAAGTCTACCAACCAACACTTCCCTGACTTACAGTACAAAAGGCACTGAGAGTTTTGGTTTCTATAATAATTCTTTCCATGCTTCCATGACTTACCAA

**L/M-CRR clone 38** sgRNA1 OT7: AC087683.5: 161702 to 162399 Homo sapiens chromosome 18.

CAGCTGTTCAGGAGGCTAAGGTGGGAGGATTGCTGGAGCCCAGGAGGTTGAGGCTGCAGTAAGCTGTGATTGCACCACTGCACTCCAGCCTGGGCAACAGAGCAAAACTTCGATTCAGGAAAAAGGAAAGGAAGGGAAGGGAGGGGAAGGGAAGGGAAGGGAAGGGAAGGGGGAAGGAAGGAAGGAAGGGAAGGAAAGAAAGGAAGGAAGGAAGAAATGGGGCACAATCAACACATTTCTAAGCCATCATTAAAACTGGTTACTAGAATAATTAAAATAGAAATTGAAACAAGGTGGCAAAAGAAGCACAAAGCCAAAACTGCAACTCCAAGGGAAAATTAAATATGGGATCATATAATAATCATTGGAAGAGCCTACCACACCAAGAAAACACCTATTTCTGTAAGTTATTTTTCTGCAGACAGGCCACCGTGGACTCTTGCAATGCAAATTGTATCCCATAATTATGAGCGTATGTTATATTCATGATTCATGAAAATCACATGGGAAGGAAAAAATCATTCCTCTCCTTTCAAAATAAGATGCTTAAGGCTTCCTTTTCTCTTATAGAGATGGCCTTGTTAATTGAATTCTGATACTCAAGTCTACCAACCAACACTTCCCTGACTTACAGTACAAAAGGCACTGAGAGTTTTGGTTTCTATAATAATTCTTTCCATGCTTCCATGACTTACCAA

**L/M-CRR clone 24** sgRNA1 OT8: AC025207.5: 154487 to 154862 Homo sapiens chromosome 8.

CTGCCCCTAAACTTCTGCCGTAGCTCCCTTTCAAGCCAGCGAATTTATTCCTTAAAACCAGAAACTGAACCTCGGCACGGGAAAGGAGTCCGCGGAGGAGCAAAACCACAGCAGAGCAAGAAGAGCTTCAGAGAGCAGCCTTCCCGGAGCACCAACTCCGTGTCGGGAGTGCAGAAACCAACAAGTGAGAGGGCGCCGCGTTCCCGGGGCGCAGCTGCGGGCGGCGGGAGCAGGCGCAGGAGGAGGAAGCGAGCGCCCCCGAGCCCCGAGCCCGAGTCCCCGAGCCTGAGCCGCAATCGCTGCGGTACTCTGCTCCGGATTCGTGTGCGCGGGCTGCGCCGAGCGCTGGGCAGGAGGCTTCGTTTTGCCCTGGTTG

**L/M-CRR clone 38** sgRNA1 OT8: AC025207.5: 154487 to 154862 Homo sapiens chromosome 8.

CTGCCCCTAAACTTCTGCCGTAGCTCCCTTTCAAGCCAGCGAATTTATTCCTTAAAACCAGAAACTGAACCTCGGCACGGGAAAGGAGTCCGCGGAGGAGCAAAACCACAGCAGAGCAAGAAGAGCTTCAGAGAGCAGCCTTCCCGGAGCACCAACTCCGTGTCGGGAGTGCAGAAACCAACAAGTGAGAGGGCGCCGCGTTCCCGGGGCGCAGCTGCGGGCGGCGGGAGCAGGCGCAGGAGGAGGAAGCGAGCGCCCCCGAGCCCCGAGCCCGAGTCCCCGAGCCTGAGCCGCAATCGCTGCGGTACTCTGCTCCGGATTCGTGTGCGCGGGCTGCGCCGAGCGCTGGGCAGGAGGCTTCGTTTTGCCCTGGTTG

**L/M-CRR clone 24** sgRNA1 OT9: AC069026.5: 82746 to 83418 Homo sapiens chromosome 10.

AGATCCAGGGCAAAGAAGGCATAATGAGTCAGATGAGTCGAAACACTCCTTCATCCTGGGTTCACTGGAGGCAGGAATCAAGAGTAAGGCAGAGATAGTGAGCACATGACTACCAGTAGTACATTTAAGGGAATAGGGTGTATGTCACTCTAAGCTCACAGGCAATTGCCTGAATAGTCCATTTAAAAGAAACAGTGGGAAAGTGGGGAGCCCAGTCTGCATGACAAAGAGATGCCTGTAAATTCCTATCTCTGGCTACCGGCTTGAGCCAGTTGGATGGATATGGTGTAGAACTAGAAACTGAATCAAGGTTGACTGAGCCCTGCTTCTGGTATGAGAAAGTTAAACTTGTATTCAAAATGGATGCCAAAGCAACATAAAATTATGAGAATTCACTGCAGTTATTACGTTTACTTTATTACTCACATAGAAAATATTACATTACACATTTATTCTATTATAATATAATAAAAAACCTTCTATCAAAGATTATGATGTACATATTAAAAATTAAAATTTTAGAAAGGCCAATAGAGAATCATGCACTTAGCTAACACAATAAAATTAACCGTTCTGCTTTCCACATTAATGGTGTAAAAGAAAAATTTGTTAGAAACAATGACCCTACATTGTGTTTTGAATATTGTTATCTGAGGGCACTCTAAAGATCTCC

**L/M-CRR clone 38** sgRNA1 OT9: AC069026.5: 82746 to 83418 Homo sapiens chromosome 10.

AGATCCAGGGCAAAGAAGGCATAATGAGTCAGATGAGTCGAAACACTCCTTCATCCTGGGTTCACTGGAGGCAGGAATCAAGAGTAAGGCAGAGATAGTGAGCACATGACTACCAGTAGTACATTTAAGGGAATAGGGTGTATGTCACTCTAAGCTCACAGGCAATTGCCTGAATAGTCCATTTAAAAGAAACAGTGGGAAAGTGGGGAGCCCAGTCTGCATGACAAAGAGATGCCTGTAAATTCCTATCTCTGGCTACCGGCTTGAGCCAGTTGGATGGATATGGTGTAGAACTAGAAACTGAATCAAGGTTGACTGAGCCCTGCTTCTGGTATGAGAAAGTTAAACTTGTATTCAAAATGGATGCCAAAGCAACATAAAATTATGAGAATTCACTGCAGTTATTACGTTTACTTTATTACTCACATAGAAAATATTACATTACACATTTATTCTATTATAATATAATAAAAAACCTTCTATCAAAGATTATGATGTACATATTAAAAATTAAAATTTTAGAAAGGCCAATAGAGAATCATGCACTTAGCTAACACAATAAAATTAACCGTTCTGCTTTCCACATTAATGGTGTAAAAGAAAAATTTGTTAGAAACAATGACCCTACATTGTGTTTTGAATATTGTTATCTGAGGGCACTCTAAAGATCTCC

**L/M-CRR clone 24** sgRNA1 OT10: AC118755.7: 154476 to 155075 Homo sapiens chromosome 17.

GGGTGACAGAGTGAGACTCCGTCTCAAAAAAAAAAAAAAAATTAGCTAGGCACGATGGCATGTACCTGTAGTGTCAGCTACTTGGGAGGCTGAGGTGAGAGGATTGGTTGATCATCCAGGAGTTCGAGGCTGCAGTGAGCCATGATCACGCCACTGCACCCCAGCCTGGGTGATAGAGAGAGGCTCTGTCTCTAAATAAAAAATAATTTGAAAATTTTTTAAAAAAGAAAATGAACCAAGGCTGCTTGAATACTTTTTCCTTTGTCAGAGTTTTTGCTTCCTGGCTCCAGCCTGCAATCTTGGCAGGCTCCTTCAGCAACTGCAACTCCTTCAGTGCCTGGCCCCTTCAGTGCACAGTGGCCAGCAATACCCAGAACCAGCAACTTCTCCCAGCACACCCCCTCCTCGGGCAGGTTTATAACAAGAGCGCCTCCAGGGAGGTTCTTCCCCATGAAGAGTTTCCCCCAGCATCCCAGAGGGCAGATCTCCAGGAGGTTCTGACAGCAGGGCACCACGGGGGCTTCTGTGAAATTCAGGGACCCTCAGCTGAGCCTTCTCTTAGCCCTAGGGGCGTGGGCTCCTCCCTGGACATTCTGTCTC

**L/M-CRR clone 38** sgRNA1 OT10: AC118755.7: 154476 to 155075 Homo sapiens chromosome 17. GGGTGACAGAGTGAGACTCCGTCTCAAAAAAAAAAAAAAAATTAGCTAGGCACGATGGCATGTACCTGTAGTGTCAGCTACTTGGGAGGCTGAGGTGAGAGGATTGGTTGATCATCCAGGAGTTCGAGGCTGCAGTGAGCCATGATCACGCCACTGCACCCCAGCCTGGGTGATAGAGAGAGGCTCTGTCTCTAAATAAAAAATAATTTGAAAATTTTTTAAAAAAGAAAATGAACCAAGGCTGCTTGAATACTTTTTCCTTTGTCAGAGTTTTTGCTTCCTGGCTCCAGCCTGCAATCTTGGCAGGCTCCTTCAGCAACTGCAACTCCTTCAGTGCCTGGCCCCTTCAGTGCACAGTGGCCAGCAATACCCAGAACCAGCAACTTCTCCCAGCACACCCCCTCCTCGGGCAGGTTTATAACAAGAGCGCCTCCAGGGAGGTTCTTCCCCATGAAGAGTTTCCCCCAGCATCCCAGAGGGCAGATCTCCAGGAGGTTCTGACAGCAGGGCACCACGGGGGCTTCTGTGAAATTCAGGGACCCTCAGCTGAGCCTTCTCTTAGCCCTAGGGGCGTGGGCTCCTCCCTGGACATTCTGTCTC
