# Supplementary material for: Identification and enrichment of human retinal organoid-derived red/green cone-competent precursors with enhanced axon dynamics
Source: Stem Cell Res Ther. 2026 Jul 4;17:336. doi: 10.1186/s13287-026-05128-9 (PMC13613627; doi:10.1186/s13287-026-05128-9)
Supplement: Supplementary file 2 [file 13287_2026_5128_MOESM2_ESM.docx]

**Data S3.**

Representative flow cytometry data showing gating strategies used to quantify D50 and D100 PR populations in L/M-CRR ROs.

FACS gating strategy for D50 L/M-CRR ROs. Debris was excluded using FSC/SSC **(Ai)**, singlets were gated using FSC-A vs FSC-H **(Aii)** and SSC-A vs SSC-H **(Aiii)**, and live cells were identified by exclusion of DAPI **(Aiv)**. **(B)** The live cell population was then subdivided into tdTomato-negative (left box) and tdTomato-positive (right box) fractions based on reporter fluorescence intensity. **(C)** Percentages of THRB2:tdTomato-positive and THRB2:tdTomato-negtaive populations within the live cell gate were 14.0% and 82.9%, respectively (orange box).

FACS gating strategy for D100 L/M-CRR ROs. Debris was excluded using FSC/SSC **(Ai)**, singlets were gated using FSC-A vs FSC-H **(Aii)** and SSC-A vs SSC-H **(Aiii)**, and live cells were identified by exclusion of DAPI **(Aiv)**. **(B)** The live cell population was then subdivided into tdTomato-positive (bottom right), eGFP-positive (top left), eGFP/tdTomato double-positive (top right), and eGFP/tdTomato double-negative (bottom left) populations. **(C)** Percentages of THRB2:tdTomato-positive, NRL:eGFP-positive, eGFP/tdTomato double-positive, and eGFP/tdTomato double-negative populations within the live cell gate were 22.8%, 17.6%, 0.2%, and 44.0%, respectively (orange box).
