# Supplementary material for: Identification and enrichment of human retinal organoid-derived red/green cone-competent precursors with enhanced axon dynamics
Source: Stem Cell Res Ther. 2026 Jul 4;17:336. doi: 10.1186/s13287-026-05128-9 (PMC13613627; doi:10.1186/s13287-026-05128-9)
Supplement: Supplementary file 3 [file 13287_2026_5128_MOESM3_ESM.docx]

Supplementary Materials for

**Identification and enrichment of integration-primed red/green cone precursors from human retinal organoids**

Praveen J. Susaimanickam *et al.*

*Corresponding author – David M. Gamm. Email: dgamm@wisc.edu

**Fig. S1. L/M-CRR hPSC line generation and validation.** **(A)** Schematic of the donor plasmid used for targeted homology-mediated CRISPR-Cas9 gene editing at the *THRB2* locus. **(B)** Graphic depicting a *tdTomato-P2A* transgene inserted 5’ to the translation initiation site (ATG) of one *THRB2* allele of the L/M-CRR line, while the other allele remains unedited. Primers that amplify DNA sequences spanning the junction between (1) the left homology arm and the tdTomato transgene (left junction primers) or (2) the left and right homology arms of *THRB2* (homology arm primers) are indicated by half arrows. **(C, D)** Agarose gel images of genomic PCR products amplified from two L/M-CRR clones (L/M-CRR C24 and L/M-CRR C38) using (C) the left junction primers (868 bp) or (D) the homology arm primers (2214 bp (edited allele) or 720 bp (unedited allele)). The parent WA09 *NRL*^+/eGFP^ line[1] shows no amplified PCR product using the left junction primers and only a 720 bp product using the homology arm primers, whereas both L/M-CRR clones also possess expected 2214 bp products. **(E)** G-banding analysis demonstrating normal karyotypes for both L/M-CRR clones.

**Fig. S2. L/M-CRR clones 24 and 38 express key pluripotency markers.** Phase-contrast (far left) and ICC (right) images of L/M-CRR hPSC colonies confirming expression of the pluripotency markers OCT3/4, SOX2, and NANOG. Scale bars: 100 µm*.*

**Fig. S3. Expression of THRB2:tdTomato in L/M-CRR ROs. (A)** ICC image of a D30 L/M-CRR RO showing THRB2:tdTomato^+^ cells predominantly along the basal neuroepithelium. **(B)** Live light microscopic **(i),** tdTomato fluorescence **(ii)**, and eGFP fluorescence **(iii)** images of D35 L/M-CRR ROs. (**C**) Representative ICC image of a D100 L/M-CRR RO showing no overlap between tdTomato (THRB2) and eGFP (NRL) immunofluorescence (white arrows point to individual tdTomato+/eGFP- cells. (**D**) Bar graph showing the percentage of FAC-sorted cells from D100 L/M-CRR ROs (>50 ROs from 4 independent differentiations) that are THRB2:tdTomato^+^ (20.9 ± 2.8), NRL:eGFP^+^ (13.5 ± 6.6), or THRB2:tdTomato/NRL:eGFP double-positive (0.2 ± 0.08). Graphs are plotted as mean ± SD. See Data S3 for gating strategies. (**E**) ICC image of a D175 L/M-CRR RO showing uniform overlap of THRB2:tdTomato expression with GNAT2 immunostaining, demonstrating that all THRB2:tdTomato+ cells are cones. Scale bars for panels A: 20 µm, B: 200 µm, C and E: 25 µm.

**Fig. S4. Absence of THRB2:tdTomato expression in non-photoreceptor cells. (A-C)** ICC images of D100 L/M-CRR ROs showing no overlap between THRB2:tdTomato (red) and markers of **(A, B)** retinal progenitors (Ki-67 and SOX2) and **(C)** retinal ganglion cells (RGCs) (SNCG). **(D)** ICC image of a D160 L/M-CRR RO showing no overlap between THRB2:tdTomato (red) and markers of RGCs and amacrine cells (Calretinin). Scale bars for panels A-D: 25 µm.

**Fig. S5. L/M-CRR ROs develop a robust outer brush border and produce THRB2:tdTomato^+^ cells that co-express L/M-Opsin and other L/M cone genes but do not co-express NRL:eGFP or S-Opsin.** **(A)** Light microscopic image of a D175 L/M-CRR RO with a uniform outer brush border indicative of an outer segment-like layer. **(B)** Representative high magnification ICC image of a D175 L/M-CRR RO immunostained for NRL:eGFP (green), THRB2:tdTomato (red), and S-Opsin (white). The white asterisks denote the two S-Opsin^+^ cones in the field, neither of which express THRB2:tdTomato. (**C**) Representative high magnification ICC image of a D175 L/M-CRR RO immunostained for NRL:eGFP (green), THRB2:tdTomato (red), and L/M-Opsin (white). The yellow asterisks denote the three L/M-Opsin^+^ cones in the field, all of which co-express THRB2:tdTomato. (**D**) Bulk RNAseq heatmap with TPM values showing the presence of L/M cone-specific genes (VOPP1, MYH4, and LMO4)[2] in the THRB2:tdTomato^+^ fraction of D100 FAC-sorted L/M-CRR ROs. Scale bars for panel A: 200 µm; B and C: 10µm.

**Fig. S6. CD166/ALCAM colocalizes with post-mitotic D50 PRs but is not expressed in D100 ROs**. ICC of D50 (top row) and D100 (bottom row) wildtype WA09 ROs showing expression of CD166/ALCAM in a subset of RCVRN^+^ photoreceptor precursors at D50 along the basal and apical aspects of the outer neuroepithelium. No CD166/ALCAM immunostaining is present in D100 ROs. Note that CD166/ALCAM^+^ cells do not express the cell proliferation marker Ki-67, indicating that they are post-mitotic. Scale bars: 200 µm.

Fig. S7. ImageJ-based quantification of CD166/ALCAM^+^ L/M cone-competent precursors in the apical or basal regions of L/M-CRR D50 ROs. ICC of L/M-CRR ROs at D50 showing cells immunostained for THRB2:tdTomato (red) and CD166/ALCAM (green). The neuroepithelium of each RO was segmented using ImageJ into two laminar regions: (1) an outermost apical region containing densely packed DAPI^+^ nuclei (white) and (2) a deeper basal region with more sparsely distributed DAPI^+^ nuclei. The yellow line demarcates the division between the apical and basal regions in the representative RO shown. The *left* *panel* shows an image of one of the 5 ROs quantified using the cell counter plugin of ImageJ. A magnified image of the boxed area without the DAPI channel is shown in the *right* *panel*. Cells were assigned a number based on their fluorescence signal and location, which were subsequently quantified and plotted in Figure 5B. Scale bars: 100 µm.

**Fig. S8. ImageJ-based quantification of PR precursor axon length.** Representative images and corresponding axon length quantifications of PR precursors from dissociated ROs derived from the WA09 *CRX*^+/tdTomato^ pan-PR reporter line[3] after **(A)** and before **(B)** CD166-mediated MACS.

**Fig. S9. RGCs do not express CD166/ALCAM in early ROs.** Low magnification ICC image (*left panel*) of a D36 RO from the E4-H7 *BRN3B*:tdTomato RGC reporter hPSC line[4] showing the presence of BRN3B:tdTomato^+^ (red) and few CD166/ALCAM^+^ cells (green) at D36 along the basal (i.e., innermost) aspect of the RO neuroepithelium. The area demarcated by the box is magnified in the *right panel*, which shows that BRN3B:tdTomato^+^ RGCs do not co-express CD166/ALCAM (*arrows*). Scale bars: 25 µm.

Table S1.

List of Primers.

| **Primers used to amplify DNA fragments for Gibson assembly of donor plasmid** | **Sequence (5’-3’)** |
| --- | --- |
| THRB2 5' homology arm-F | ATCGATAAGCTTGATATCGAATTCAACAACAGATTCAACCACC |
| THRB2 5' homology arm-R | GCCCTTGCTCACCATTTTGTTTCCCTGGTTCAGTTTCTAG |
| tdTomato-F | GAACCAGGGAAACAAAATGGTGAGCAAGGGCGAGG |
| tdTomato_P2A-R | CATCTCCGGCTTGTTTCAGCAGAGAGAAGTTTGTTGCGCCGGATCCCTTGTACAGCTCGTCCATGC |
| THRB2 3' homology arm_P2A-F | CTTCTCTCTGCTGAAACAAGCCGGAGATGTCGAAGAGAATCCTGGACCGATGAACTACTGTATGCAAGAAATATATG |
| THRB2 3'homology arm-R | GGAGCTCCACCGCGGTGGCGGCCGCTACATCTTACAGTTTGACATGG |
| **Genotyping primers** |  |
| Left Junction-F | GAAAATAATCATGCGCAAGG |
| Left Junction-R | ACCTTGAAGCGCATGAACTC |
| Homology arm-F | GGATGGCAGAAGAGCTCAAA |
| Homology arm-R | GCAACCCCTCTTACTGCCG |
| **Off-target (OT) screening primers** |  |
| OT1-F | GTGGTTATTGAAGGAGTCCTCC |
| OT1-R | ACATGGCACCCTTCTAAAGGG |
| OT2-F | CTCACTGCAACCTTCACTTCC |
| OT2-R | ACCGTTCCAGGACTTTGAGG |
| OT3-F | GGGTAGACACCAAGGAGCGC |
| OT3-R | CCAAGGGGCAGCTATGAGTG |
| OT4-F | TGTCACCCAGGAGTTCAATGG |
| OT4-R | CGATCTCAGCTCACTGCAACC |
| OT5-F | GGGTGTGATTAGCTGTGTGTCG |
| OT5-R | TCAGCTGCTCTGTTTCTCAGG |
| OT6-F | ACATCCGAATCCATCAGTGGC |
| OT6-R | CCCTTCAGGATGACACAGAC |
| OT7-F | CAGCTGTTCAGGAGGCTAAGG |
| OT7-R | TTGGTAAGTCATGGAAGCATGG |
| OT8-F | CTGCCCCTAAACTTCTGCCG |
| OT8-R | CAACCAGGGCAAAACGAAGC |
| OT9-F | AGATCCAGGGCAAAGAAGGC |
| OT9-R | GGAGATCTTTAGAGTGCCCTCAG |
| OT10-F | GGGTGACAGAGTGAGACTCC |
| OT10-R | GAGACAGAATGTCCAGGGAGG |
| **qPCR primers** |  |
| THRB2-F | GGGTTACAGCGGTTGCGATGCT |
| THRB2-R | GCTGATTCACTGCCCAGGCCTG |
| GNAT2-F | GCCTGCTTCGAGAGAGCTG |
| GNAT2-R | GCACATCTTGCTCACTAGGGA |
| ARR3-F | AGGAAAGCCCTGTGGGATTGACTT |
| ARR3-R | AACCAGCCGCACATAGTCTCTCTT |
| GNGT2-F | TCTCAGCGAGAAGGACCTGT |
| GNGT2-R | AGGATCGTTTCCTGCTTGGG |
| GAPDH-F | CAACGGATTTGGTCGTATTGG |
| GAPDH-R | GCAACAATATCCACTTTACCAGAGTTAA |
| YWHAZ-F | ACTTTTGGTACATTGTGGCTTCAA |
| YWHAZ-R | CCGCCAGGACAAACCAGTAT |

Table S2.

Top 10 off target (OT) sites for sgRNA.

| **sgRNA sequence:** GTAAACTAGAAACTGAACCAGGG | | | | | | | |
| --- | --- | --- | --- | --- | --- | --- | --- |
| **Guide ID** | **Chrom-osome** | **Strand** | **OT sequence** | **Mismatch count** | **mitOff-target score** | **Locus description** |  |
| sgRNA OT1 | chr5 | + | TAAAAATAGAAACTGAACCGGGG | 4 | 0.421955546 | exon:SNX24 |  |
| sgRNA OT2 | chr1 | + | GAAAATTAGAGACTGAACCGGGG | 4 | 0.234185328 | intron:EDARADD |  |
| sgRNA OT3 | chr8 | + | TTTAACTAGTAACAGAACCAAGG | 4 | 0.20338988 | intron:PVT1 |  |
| sgRNA OT4 | chr17 | - | AGAAAATAGAAACTGAAACATGG | 4 | 0.187751667 | exon:RP11-192H23.6/ SGK494/RP11-192H23.4 |  |
| sgRNA OT5 | chr1 | + | GTAAGCAAGTGACTGAACCAAGG | 4 | 0.476527186 | intergenic:RP1-35C21.2- RP11-63B19.1 |  |
| sgRNA OT6 | chr5 | - | GGAAACTAAAAATTAAACCAGGG | 4 | 0.095256253 | intergenic:RBM27-CTC-359M8.1 |  |
| sgRNA OT7 | chr18 | + | TTAAAATAGAAATTGAAACAAGG | 4 | 0.072659895 | intergenic:AC011225.1-PIK3C3 |  |
| sgRNA OT8 | chr8 | + | TAAAACCAGAAACTGAACCTCGG | 4 | 0.476356426 | exon:SDC2 |  |
| sgRNA OT9 | chr10 | + | TAGAACTAGAAACTGAATCAAGG | 4 | 0.305988667 | intergenic:SNORD2-MIR3924 |  |
| sgRNA OT10 | chr17 | + | TTAAAAAAGAAAATGAACCAAGG | 4 | 0.240377365 | intron:STX8 |  |

Table S3.

List of antibodies used for ICC and MACS.

| **Antibody** | **Host** | **Company** | **Catalog Number** | **Dilution** |
| --- | --- | --- | --- | --- |
| ALCAM/CD166 | Rabbit | R&D Systems | MAB6563 | 1:100 |
| GFP | Chicken | Invitrogen | A10262 | 1:250 |
| GNAT2 | Rabbit | LSBio | LS-C321680 | 1:100 |
| L/M-Opsin | Rabbit | Millipore | AB5405 | 1:500 |
| Recoverin | Rabbit | Proteintech | 10073-1-AP | 1:1000 |
| S-Opsin | Rabbit | Millipore | AB5407 | 1:500 |
| tdTomato | Goat | SICGEN | AB8181-200 | 1:300 |
| OCT3/4 | Mouse | Santa Cruz | SC-5279 | 1:100 |
| SOX2 | Goat | R&D | AF2018 | 1:100 |
| NANOG | Goat | R&D | AF1997 | 1:100 |
| Ki67 | Rabbit | Abcam | AB15580 | 1:100 |
| Ki67 | Rabbit | Abcam | AB92742 | 1:100 |
| SNCG | Mouse | Abnova | H00006623-M01 | 1:500 |
| Calretinin | Rabbit | Millipore | AB5054 | 1:500 |
| CD166-PE | Human cell line | Miltenyi Biotec | 130-118-349 | 1:50 |
| CD274-PE | Human cell line | Miltenyi Biotec | 130-122-815 | 1:50 |
| CD52-PE | Human cell line | Miltenyi Biotec | 130-123-743 | 1:50 |
| CD166-Biotin | Human cell line | Miltenyi Biotec | 130-126-100 | 1:50 |
| Anti-Rabbit 555 | Donkey | Life Technologies | A10040 | 1:500 |
| Anti-Chicken 488 | Donkey | Jackson Immuno | 703-545-155 | 1:500 |
| Anti-Goat 555 | Donkey | Life Technologies | A11056 | 1:500 |
| Anti-Mouse 488 | Donkey | Life Technologies | A21202 | 1:500 |
| Anti-Goat 633 | Donkey | Life Technologies | A21082 | 1:500 |
| Anti-Mouse 647 | Donkey | Life Technologies | A31571 | 1:500 |
| Anti-Rabbit 633 | Donkey | Life Technologies | A31573 | 1:500 |

Table S4.

Top 10 biological processes associated with genes from Cluster 2 in Figure 4D.

| GO term | p-value | q-value | Genes found in Cluster 2 |
| --- | --- | --- | --- |
| Inorganic Cation Transmembrane Transport (GO:0098662) | 2.42E-05 | 3.38E-02 | *TRPC7, SLC6A19, TRPC5, KCNC2, KCNK12, SLC41A2, SLC39A11, ATP2A3, ATP2A1, HTR3A, SLC6A4, CACNA1G, SLC6A5, KCNS3, KCNQ5, SCN1B* |
| Axon Guidance (GO:0007411) | 1.27E-04 | 5.89E-02 | *ALCAM, SEMA6B, RET, NELL2, EPHA7, NRP2, UNC5B, NRXN1, SCN1B, CDK5R1* |
| Neuromuscular Process (GO:0050905) | 2.20E-04 | 6.17E-02 | *CHRNA1, GCH1, NRXN1, HOXA1* |
| Cellular Response to Forskolin (GO:1904322) | 2.84E-04 | 6.17E-02 | *ADCY2, ADCY5, SLC5A5* |
| Response to Forskolin (GO:1904321) | 2.84E-04 | 6.17E-02 | *ADCY2, ADCY5, SLC5A5* |
| Adenylate Cyclase-Modulating G Protein-Coupled Receptor Signaling Pathway (GO:0007188) | 3.11E-04 | 6.17E-02 | *PTGFR, ADGRG4, GIPR, GRM8, GLP2R, FFAR4, PTH2R, ADCY2, ADRA2A, TSHR, ADCY5* |
| Desmosome Organization (GO:0002934) | 4.01E-04 | 6.17E-02 | *RCAN1, PKP2, SNAI2* |
| Regulation of Endothelial Cell Migration (GO:0010594) | 4.20E-04 | 6.17E-02 | *ACVRL1, DLL4, NRP2, BMPER, EGF, SVBP, STC1* |
| Modulation of Chemical Synaptic Transmission (GO:0050804) | 4.61E-04 | 6.17E-02 | *RIMS1, GRID2, ADIPOQ, GRM8, NTF3, NGF, GRIA3, CNTNAP4* |
| Regulation of Cell Migration (GO:0030334) | 4.87E-04 | 6.17E-02 | *PTPRT, RET, ACVRL1, SEMA6B, EGF, TNNC1, ADIPOQ, STC1, LAMB1, PODN, ADRA2A, BMPER, PDGFD, DLC1, NTF3, TP53INP1, SNAI2, CCL18* |

Table S5.

Top 10 biological processes associated with genes upregulated in D50 L/M cone-competent precursors.

| GO term | p-value | q-value | Genes found in D50 L/M cone-competent precursors |
| --- | --- | --- | --- |
| Regulation of Cell Migration (GO:0030334) | 3.54E-13 | 1.18E-09 | *RET, ITGB1, ACVRL1, PTPRT, IFITM1, SERPINE2, KIF14, PTPRK, CLDN1, ROBO1, AMOT, FGF9, MDK, CYP1B1, PLXNC1, EPHB2, TMSB10, SEMA6B, PLEKHG3, PDGFRB, EPHA4, IGFBP5, TNNC1, ADIPOQ, EMP2, ADRA2A, SAP30, CLDN5, MMP14, TMEFF2, SFRP1, SFRP2, RAP2B, NAV3, TMSB15B, SPATA13, MMRN2, PECAM1, MYADM, CDH13, ITGA6, FBN2, SYNPO2, LEF1, STC1, CXCR4, PTN, THBS1, EGFR, RELN, PDGFD, NTF3, PDPN, GCNT2, PLXNA2, FLNA, CTNNA2, SLIT2, CCL18, DRD2, SRGAP2, RFFL, SRGAP1, EGF, SULF1, TBX5, PODN, TMEM196, STK26, DLC1, SNAI2, SPRY2, EPPK1* |
| Nervous System Development (GO:0007399) | 3.23E-12 | 5.38E-09 | *PPP1R17, GRIK1, ROBO1, FGF9, ZIC2, MDK, ZIC3, BOC, DNER, PLXNC1, EPHB2, SOX6, SOX4, DACT1, CHST8, SEMA6B, RBFOX1, TRPC5, RBFOX2, SEMA6A, SOX11, PAX6, NAV1, PROX1, GFRA2, VCAN, NAV3, B3GNT5, DCX, TAGLN3, NLGN1, SHOX2, LPAR1, NCAPG, CXCR4, PTN, ASCL1, UGT8, NHLH2, RELN, EFNB3, ACVR1C, PTPRZ1, SLITRK1, NHLH1, NTF3, PLXNA2, ST8SIA4, SPOCK1, IGF2BP3, DRD2, HES5, MPP2, FZD5, BDNF, CNTN6, ZBTB16, FN1, LSAMP, NR2F2, ZIC5, MERTK, NELL1, SDK1, FGF14, MAB21L2, SP8, SERPINI1, CNTN4, NES, MDGA1* |
| Axon Guidance (GO:0007411) | 5.90E-12 | 6.56E-09 | *EPHB6, RET, CYFIP1, NOTCH3, NRP1, NRP2, NTN4, EFNA4, GLI2, EFNB2, ALCAM, EFNB3, RELN, BOC, PLEKHG4B, FYN, SLIT2, EPHB2, SEMA6B, EPHA4, EPHA7, SEMA6A, CNTN5, UNC5B, BDNF, CNTN6, WNT5A, POU4F2, NELL2, KIAA1755, KLF7, GAP43, NFIB, CNTN2, CNTN4* |
| Axonogenesis (GO:0007409) | 8.49E-11 | 7.07E-08 | *EPHB6, RET, CYFIP1, NRP1, NOTCH3, NRP2, NTN4, EFNA4, GLI2, EFNB2, RELN, EFNB3, SLITRK2, SLITRK1, SLITRK4, PLEKHG4B, BOC, SLITRK5, FYN, CTNNA2, SLIT2, EPHB2, DRD2, SEMA6B, EPHA4, EPHA7, SEMA6A, CNTN5, RIPK2, BDNF, CNTN6, WNT5A, ISL1, POU4F2, KIAA1755, KLF7, S100A6, CNTN2, CNTN4* |
| Cell-Cell Adhesion via Plasma-Membrane Adhesion Molecules (GO:0098742) | 1.96E-10 | 1.31E-07 | *ITGB1, PTPRT, NLGN1, TENM3, AMIGO2, CELSR2, CLDN1, CDH8, FGFRL1, ROBO1, CDH6, CDH4, ALCAM, SLITRK2, CDH20, SLITRK1, SLITRK5, GPC4, TGFB2, GRID2, CNTN6, PCDHGC3, CLDN5, SDK1, CLDN10, LRFN5, SELENOP, CDHR3, PECAM1, CNTN2, CDH13, DSG2, CNTN4, NECTIN3, CDH18* |
| Neuron Projection Guidance (GO:0097485) | 2.67E-10 | 1.49E-07 | *EPHB6, RET, CYFIP1, NOTCH3, NRP1, NRP2, NTN4, EFNA4, GLI2, EFNB2, EFNB3, RELN, BOC, PLEKHG4B, FYN, SLIT2, EPHB2, SEMA6B, EPHA4, EPHA7, SEMA6A, CNTN5, BDNF, CNTN6, WNT5A, POU4F2, KIAA1755, KLF7, CNTN2, CNTN4* |
| Negative Regulation of Cell Migration (GO:0030336) | 1.57E-09 | 7.46E-07 | *ACVRL1, PTPRT, IFITM1, STC1, PTPRK, THBS1, ROBO1, DLL4, GJA1, ADGRG1, CYP1B1, SLIT2, DRD2, SRGAP2, SRGAP1, EPHA4, IGFBP5, ADIPOQ, NR2F2, PODN, SULF1, TBX5, TMEM196, SAP30, CLDN5, SFRP1, TMEFF2, SFRP2, RAP2B, NAV3, STK26, DLC1, MMRN2, EPPK1, PHLDB2* |
| Skeletal System Development (GO:0001501) | 2.14E-09 | 8.93E-07 | *DLX1, COL13A1, SHOX2, STC1, PCSK5, GLI2, DLL3, GJA1, FRZB, TRPS1, SH3PXD2B, PLS3, ITGB8, SOX9, SOX6, HES5, TGM2, TGFB2, ZBTB16, WNT5A, SULF1, TBX4, TBX3, SULF2, MMP14, VCAN, BMP1, COL1A2, COL2A1, ALPL, CD44, FBN1* |
| Negative Regulation of Cell Motility (GO:2000146) | 4.38E-09 | 1.62E-06 | *PTPRT, IFITM1, STC1, FBLN1, PTPRK, ROBO1, CYP1B1, SLIT2, DRD2, SRGAP2, SRGAP1, EPHA4, IGFBP5, ADIPOQ, PODN, SULF1, TBX5, TMEM196, SAP30, CLDN5, SFRP1, TMEFF2, SFRP2, RAP2B, NAV3, STK26, DLC1, MMRN2, EPPK1* |
| Central Nervous System Development (GO:0007417) | 5.66E-09 | 1.89E-06 | *PPP1R17, AMIGO2, NCAPG, CXCR4, GRIK1, SOX2, C3, NHLH2, UGT8, ADGRG1, RELN, ZIC2, PTPRZ1, ZIC3, NHLH1, DNER, SPOCK1, SOX9, SOX6, BRINP3, SOX4, GPR158, HES5, CHST8, SEMA6B, EPHA7, CNTN5, CNTN6, ZBTB16, SOX11, PAX6, PROX1, ZIC5, SHROOM4, SLC8A3, VCAN, NFIB, B3GNT5, DCX, SP8, TAGLN3, SERPINI1, CNTN4, NES, MDGA1* |

Table S6.

Top 10 biological processes associated with genes upregulated in D100 L/M cone-competent precursors.

| GO term | p-value | q-value | Genes found in D100 L/M cone-competent precursors |
| --- | --- | --- | --- |
| Monoatomic Cation Transmembrane Transport (GO:0098655) | 3.4E-08 | 7.80E-05 | *SLC24A4, SLC24A3, KCNC4, KCNA3, KCNA5, CACNA1D, SLC8A1, KCNV2, KCNT1, SLC39A5, KCNN3, SCN5A, SCN3B, ASIC2, TRPM6, KCNF1, KCND3, SLC30A2, SLC6A15, KCNB2, TRPV2, KCNK15, SLC6A11, GRIN2B, SLC6A6, CACNB2, SLC4A9, KCNQ1, KCNQ3, SLC29A4, CNGB3, SCN2A, SLC24A5, CNGB1, HCN1* |
| Action Potential (GO:0001508) | 7.50E-08 | 8.60E-05 | *KCNF1, KCND3, KCNE4, KCNIP2, KCNB2, KCNC4, KCNA3, KCNA5, ANK3, KCNV2, KCNQ1, DMD, SCN5A, SCN3B, SCN2A* |
| Visual Perception (GO:0007601) | 6.50E-07 | 5.00E-04 | *RD3, CABP4, GUCA1B, SLC4A10, GUCA1C, DHRS3, GNAT2, RDH12, RGS9BP, PRPH2, GRM6, RDH10, KIFC3, EYS, NXNL2, GPR179, CNGB1* |
| Cardiac Muscle Cell Action Potential (GO:0086001) | 1.50E-06 | 8.90E-04 | *KCND3, KCNIP2, KCNQ1, CACNA1D, DMD, SCN5A, SCN3B, SCN2A, FGF12, SLC8A1* |
| Inorganic Cation Transmembrane Transport (GO:0098662) | 3.50E-06 | 1.60E-03 | *SLC24A4, SLC24A3, KCNC4, KCNA3, KCNA5, CACNA1D, SLC8A1, KCNV2, KCNT1, SLC39A5, KCNN3, SCN5A, SCN3B, ASIC2, TRPM6, KCNF1, KCND3, SLC30A2, SLC6A15, KCNB2, TRPV2, KCNK15, SLC6A11, SLC6A6, CACNB2, SLC4A9, KCNQ1, KCNQ3, SCN2A, SLC24A5, HCN1* |
| Potassium Ion Transmembrane Transport (GO:0071805) | 8.70E-06 | 3.35E-03 | *KCNF1, SLC24A4, SLC24A3, SLC12A5, KCNE4, KCND3, KCNB2, KCNC4, KCNA3, KCNK15, KCNA5, KCNV2, KCNT1, KCNQ1, KCNQ3, KCNN3, SLC12A8, HCN1* |
| Sensory Perception of Light Stimulus (GO:0050953) | 1.40E-05 | 4.65E-03 | *RD3, CABP4, GUCA1B, SLC4A10, GUCA1C, USH2A, DHRS3, GNAT2, RDH12, PRPH2, RDH10, KIFC3, NXNL2, GPR179, CNGB1* |
| Potassium Ion Transport (GO:0006813) | 1.60E-05 | 4.65E-03 | *KCNF1, SLC24A4, SLC24A3, KCND3, KCNIP2, KCNB2, KCNC4, KCNA3, KCNK15, KCNA5, KCNV2, KCNT1, KCNQ1, KCNQ3, KCNN3, CNGB1, HCN1* |
| Regulation of Heart Rate by Cardiac Conduction (GO:0086091) | 3.20E-05 | 8.27E-03 | *CACNB2, KCNE4, KCND3, KCNQ1, CACNA1D, KCNA5, SCN5A, SCN3B, HCN1* |
| SA Node Cell Action Potential (GO:0086015) | 3.90E-05 | 8.87E-03 | *CACNA1D, KCNA5, SCN5A, SCN3B* |

Table S7.

Total percentages of THRB2:tdTomato+ L/M cone-competent precursors co-expressing CD166/ALCAM in five individual ROs.

| **RO #** | **Percentage** |
| --- | --- |
| RO1 | 59.58 |
| RO2 | 60.94 |
| RO3 | 61.61 |
| RO4 | 54.24 |
| RO5 | 63.82 |
| **Mean ± SD** | **60.04 ± 3.58** |

Table S8.

Percentages of THRB2:tdTomato+ L/M cone-competent precursors co-expressing CD166/ALCAM within the apical or basal neuroepithelial regions of five individual ROs.

| **RO #** | **Apical region** | **Basal region** |
| --- | --- | --- |
| RO1 | 47.41 | 71.75 |
| RO2 | 38.86 | 83.01 |
| RO3 | 40.07 | 83.14 |
| RO4 | 39.01 | 69.46 |
| RO5 | 43.04 | 84.61 |
| **Mean ± SD** | **41.68 ± 3.61** | **78.40 ± 7.18** |

Data S1. (Separate file). Annotated sequence of the modified and unmodified THRB2 locus.

Data S2. (Separate file). Confirmed Sanger sequences of the top 10 predicted off-target sites in L/M-CRR clones 24 and 38 showing the absence of indels.

Data S3. (Separate file). Representative flow cytometry data showing gating strategies used to quantify D50 and D100 PR populations in L/M-CRR ROs.

**References**

1. Phillips MJ, Capowski EE, Petersen A, Jansen AD, Barlow K, Edwards KL, et al. Generation of a rod-specific NRL reporter line in human pluripotent stem cells. Sci Rep. Nature Publishing Group; 2018;8:2370. https://doi.org/10.1038/s41598-018-20813-3

2. Peng Y-R, Shekhar K, Yan W, Herrmann D, Sappington A, Bryman GS, et al. Molecular Classification and Comparative Taxonomics of Foveal and Peripheral Cells in Primate Retina. Cell. Elsevier; 2019;176:1222-1237.e22. https://doi.org/10.1016/j.cell.2019.01.004

3. Phillips MJ, Jiang P, Howden S, Barney P, Min J, York NW, et al. A novel approach to single cell RNA-seq analysis facilitates in silico gene reporting of human pluripotent stem cell-derived retinal cell types. Stem Cells. 2018;36:313–24. https://doi.org/10.1002/stem.2755

4. Sluch VM, Chamling X, Liu MM, Berlinicke CA, Cheng J, Mitchell KL, et al. Enhanced Stem Cell Differentiation and Immunopurification of Genome Engineered Human Retinal Ganglion Cells. Stem Cells Transl Med. 2017;6:1972–86. https://doi.org/10.1002/sctm.17-0059
