# Supplementary material for: Identification and enrichment of human retinal organoid-derived red/green cone-competent precursors with enhanced axon dynamics
Source: Stem Cell Res Ther. 2026 Jul 4;17:336. doi: 10.1186/s13287-026-05128-9 (PMC13613627; doi:10.1186/s13287-026-05128-9)
Supplement: Supplementary file 4 [file 13287_2026_5128_MOESM4_ESM.docx]

**Data S1.**

Annotated sequence of the modified and unmodified *THRB2* locus.

***THRB* NCBI Gene ID:** 7068

***THRB2* Ensemble ID:** ENST00000280696.9 THRB-201

***THRB2* GenBank CDS**: OQ406274.1

**THRB2 UniProt ID**: P10828-2

**Annotation based on THRB NCBI Reference Sequence: NC_000003.12**

**Original *THRB2* locus:**

*THRB2* 5’ upstream sequence (1-330113)

gccgggcaaagaaggccgagacgtgctcctggaagactcgccctccggtcccggtgtcactcgttccccattctttcctcttctccaactagagtaatgacgcccaaacccgcacctatgcgcgcaggcaatcgggtgggtggacgcgcggccaccaaacccacagcaccgtcaccaaccctgggagggcacg……………acattaaatcttgaaaataatctcaaaagcagatttgaatgggaataaagtggtaagtgtttacagttttccacgcactactgatcagagtactgttaagcaccaacagaaaaggaaagctgctatataataggggtattcctcatataggggcattggcatacagtctctcatgcctttataaagaaaacataaattacagaattgaacagttaatgtaagctttaggtctcatgtagattagtgcttatgaagtagaaaataatcatgcgcaaggaacagaataaaactcacttgcagacatcagcagcattttcaaccgtgtacttccttggcatgttttaaaaaccagcgggacctatgtgtcatgggaagatggttggatcctagtatgaaaacccagtcagtaacattcaacaacagattcaaccaccaatatttgcacagctagattaatgttttggagtcagtcagtcatcaggctcattactactggattttctctggttcatgctaataaactagattattgattttaggattttctttgtatccaaggatattctctaagatgtctttataatgtttcatgcttaagctgtcaaccaaaatgacataacaatgagtaatcgttttataatcatgttgaaaggggttaaaaacagcatataaaatgcatcttagcagcttcagagtaacattctcgctgtcacttccctatctgggtggaggtcattcctacctgcctgccatcttccccaggaattaactagctgcatgcacagcttaagacctttgatagtcataaatctgctttccaatatgtttggatgg

*THRB2* EXON 1 (330114-330682)

THRB2 Translation initiation site

cagaagagctcaaaagacacctcggtctaaataggattttagcacatcaggtgctattacaaagtaaagcaggctgggttgctgtgttggtggtggtgtttattcatcttcccttcgcgtttcatgtgtatgtatgtatgcttctctgcgtatatgcccagctctcagggtgtatggggctggagaatgcatgcgtagactgtgtgtatatgtaaactagaaactgaaccagggaaacaaaATGAACTACTGTATGCAAGAAATATATGAAGTGCACCCAGCTGCCGGCAGCAATTGCTACATGCAGTCCACTGATTATTACGCGTATTTTGAAGATAGTCCGGGTTACAGCGGTTGCGATGCTCAGGCTGTGCCCAGTAACAACATATATATGGAACAGGCCTGGGCAGTGAATCAGCCTTATACCTGTAGTTACCCTGGAAACATGTTTAAAAGCAAGGACTCTGACTTGGACATGGCCCTGAATCAATACAGCCAACCTGAATATTTCACTGAAGAAAAGCCTACTTTTTCTCAAGTGCAGTCGCCATCGTATTCTCAAAAAAAAG

*THRB2* INTRON 1 (330683-343218)

gtagggaccattttgcttaaatttgttcaaagtttcactgtttgttttcgtttcattttaaagttacagtttacatcagcttcgtcaaatctttgaatttgaacgctaaatatttgaaattaaaaacggcagtaagaggggttgctaggtgattgaaggactctgtgaacatcaagatttccaagcactttttttcttcatctttgaaagctggagtgagttgcctttctgtattttgcatgtgtgtgtatatattgcccaaagtgactattgttggcttctttgcgaaagaaaatatgtgaaaggtttccttctggcgggtaggtgttgtggtgtttgaagtgagataattctctctagttcaggttcagattaaaaactcatttgacttttccttggcacttcattttgaaagtttattttagttgactagttaactagtaaaatatttagtgatttagtgatttctttaacagtactatactaatgttgatttgctaggatctgtaaatgttcattttaatgtatttatttatccatgtaaacagtaaagaatctatttgtaaccatgtcaaactgtaagatgtaaaactcaaaaactgagaaactttactttgctattttgtccctaaagttgaacatgtctgtaatcatatcaagtgaattctctttatgaaagcaagtgaaacacacttcaaaatagccaatattaaaaccagtacgatatccatttatcttaatagacactgtggctttataatgctatttaaatttctgtcaatagaacatttcttgtattactgaaaaataagatactgaaaaataaaataagggaggcaaacaggtagaacattaagtagcaactgccagataggaatgcttttgaagatagtgtaaatgctagtgcagatatgccagaatctcatgcttttgtatataatctataggatattgcatagagaaccaaatttacgagtttctataa…………ctgaccccaggtgatccacctgcctgggcctcccaaagtgctgggattataggtgtgagctatttctaaggcattctgaaatcctgggcctatgttaactctatgagtataaaattcttaccgtttggtttcactaagtctttactgttgttacctcttcctggcaagccaattttgtctctggcctagcaaccttagctatagtgtctctttgacgttaacatattattttttaatattccttcttttatttccttcacag

**Modified *THRB2* locus:**

5’ homology arm and 3’homology arm - underlined, tdTomato Translation initiation

site, tdTomato sequence, P2A sequence, THRB2 Translation initiation site

..acattaaatcttgaaaataatctcaaaagcagatttgaatgggaataaagtggtaagtgtttacagttttccacgcactactgatcagagtactgttaagcaccaacagaaaaggaaagctgctatataataggggtattcctcatataggggcattggcatacagtctctcatgcctttataaagaaaacataaattacagaattgaacagttaatgtaagctttaggtctcatgtagattagtgcttatgaagtagaaaataatcatgcgcaaggaacagaataaaactcacttgcagacatcagcagcattttcaaccgtgtacttccttggcatgttttaaaaaccagcgggacctatgtgtcatgggaagatggttggatcctagtatgaaaacccagtcagtaacattcaacaacagattcaaccaccaatatttgcacagctagattaatgttttggagtcagtcagtcatcaggctcattactactggattttctctggttcatgctaataaactagattattgattttaggattttctttgtatccaaggatattctctaagatgtctttataatgtttcatgcttaagctgtcaaccaaaatgacataacaatgagtaatcgttttataatcatgttgaaaggggttaaaaacagcatataaaatgcatcttagcagcttcagagtaacattctcgctgtcacttccctatctgggtggaggtcattcctacctgcctgccatcttccccaggaattaactagctgcatgcacagcttaagacctttgatagtcataaatctgctttccaatatgtttggatggcagaagagctcaaaagacacctcggtctaaataggattttagcacatcaggtgctattacaaagtaaagcaggctgggttgctgtgttggtggtggtgtttattcatcttcccttcgcgtttcatgtgtatgtatgtatgcttctctgcgtatatgcccagctctcagggtgtatggggctggagaatgcatgcgtagactgtgtgtatatgtaaactagaaactgaaccagggaaacaaaATGGTGAGCAAGGGCGAGGAGGTCATCAAAGAGTTCATGCGCTTCAAGGTGCGCATGGAGGGCTCCATGAACGGCCACGAGTTCGAGATCGAGGGCGAGGGCGAGGGCCGCCCCTACGAGGGCACCCAGACCGCCAAGCTGAAGGTGACCAAGGGCGGCCCCCTGCCCTTCGCCTGGGACATCCTGTCCCCCCAGTTCATGTACGGCTCCAAGGCGTACGTGAAGCACCCCGCCGACATCCCCGATTACAAGAAGCTGTCCTTCCCCGAGGGCTTCAAGTGGGAGCGCGTGATGAACTTCGAGGACGGCGGTCTGGTGACCGTGACCCAGGACTCCTCCCTGCAGGACGGCACGCTGATCTACAAGGTGAAGATGCGCGGCACCAACTTCCCCCCCGACGGCCCCGTAATGCAGAAGAAGACCATGGGCTGGGAGGCCTCCACCGAGCGCCTGTACCCCCGCGACGGCGTGCTGAAGGGCGAGATCCACCAGGCCCTGAAGCTGAAGGACGGCGGCCACTACCTGGTGGAGTTCAAGACCATCTACATGGCCAAGAAGCCCGTGCAACTGCCCGGCTACTACTACGTGGACACCAAGCTGGACATCACCTCCCACAACGAGGACTACACCATCGTGGAACAGTACGAGCGCTCCGAGGGCCGCCACCACCTGTTCCTGGGGCATGGCACCGGCAGCACCGGCAGCGGCAGCTCCGGCACCGCCTCCTCCGAGGACAACAACATGGCCGTCATCAAAGAGTTCATGCGCTTCAAGGTGCGCATGGAGGGCTCCATGAACGGCCACGAGTTCGAGATCGAGGGCGAGGGCGAGGGCCGCCCCTACGAGGGCACCCAGACCGCCAAGCTGAAGGTGACCAAGGGCGGCCCCCTGCCCTTCGCCTGGGACATCCTGTCCCCCCAGTTCATGTACGGCTCCAAGGCGTACGTGAAGCACCCCGCCGACATCCCCGATTACAAGAAGCTGTCCTTCCCCGAGGGCTTCAAGTGGGAGCGCGTGATGAACTTCGAGGACGGCGGTCTGGTGACCGTGACCCAGGACTCCTCCCTGCAGGACGGCACGCTGATCTACAAGGTGAAGATGCGCGGCACCAACTTCCCCCCCGACGGCCCCGTAATGCAGAAGAAGACCATGGGCTGGGAGGCCTCCACCGAGCGCCTGTACCCCCGCGACGGCGTGCTGAAGGGCGAGATCCACCAGGCCCTGAAGCTGAAGGACGGCGGCCACTACCTGGTGGAGTTCAAGACCATCTACATGGCCAAGAAGCCCGTGCAACTGCCCGGCTACTACTACGTGGACACCAAGCTGGACATCACCTCCCACAACGAGGACTACACCATCGTGGAACAGTACGAGCGCTCCGAGGGCCGCCACCACCTGTTCCTGTACGGCATGGACGAGCTGTACAAGGGATCCGGCGCAACAAACTTCTCTCTGCTGAAACAAGCCGGAGATGTCGAAGAGAATCCTGGACCGATGAACTACTGTATGCAAGAAATATATGAAGTGCACCCAGCTGCCGGCAGCAATTGCTACATGCAGTCCACTGATTATTACGCGTATTTTGAAGATAGTCCGGGTTACAGCGGTTGCGATGCTCAGGCTGTGCCCAGTAACAACATATATATGGAACAGGCCTGGGCAGTGAATCAGCCTTATACCTGTAGTTACCCTGGAAACATGTTTAAAAGCAAGGACTCTGACTTGGACATGGCCCTGAATCAATACAGCCAACCTGAATATTTCACTGAAGAAAAGCCTACTTTTTCTCAAGTGCAGTCGCCATCGTATTCTCAAAAAAAAGgtagggaccattttgcttaaatttgttcaaagtttcactgtttgttttcgtttcattttaaagttacagtttacatcagcttcgtcaaatctttgaatttgaacgctaaatatttgaaattaaaaacggcagtaagaggggttgctaggtgattgaaggactctgtgaacatcaagatttccaagcactttttttcttcatctttgaaagctggagtgagttgcctttctgtattttgcatgtgtgtgtatatattgcccaaagtgactattgttggcttctttgcgaaagaaaatatgtgaaaggtttccttctggcgggtaggtgttgtggtgtttgaagtgagataattctctctagttcaggttcagattaaaaactcatttgacttttccttggcacttcattttgaaagtttattttagttgactagttaactagtaaaatatttagtgatttagtgatttctttaacagtactatactaatgttgatttgctaggatctgtaaatgttcattttaatgtatttatttatccatgtaaacagtaaagaatctatttgtaaccatgtcaaactgtaagatgtaaaactcaaaaactgagaaactttactttgctattttgtccctaaagttgaacatgtctgtaatcatatcaagtgaattctctttatgaaagcaagtgaaacacacttcaaaatagccaatattaaaaccagtacgatatccatttatcttaatagacactgtggctttataatgctatttaaatttctgtcaatagaacatttcttgtattactgaaaaataagatactgaaaaataaaataagggaggcaaacaggtagaacattaagtagcaactgccagataggaatgcttttgaagatagtgtaaatgctagtgcagatatgccagaatctcatgcttttgtatataatctataggatattgcatagagaaccaaatttacgagtttctataa..
